# Representation of multimorbidity and frailty in the development and validation of kidney failure prognostic prediction models: a systematic review

**DOI:** 10.1186/s12916-024-03649-9

**Published:** 2024-10-11

**Authors:** Heather Walker, Scott Day, Christopher H. Grant, Catrin Jones, Robert Ker, Michael K. Sullivan, Bhautesh Dinesh Jani, Katie Gallacher, Patrick B. Mark

**Affiliations:** 1 School of Cardiovascular and Metabolic Health, University of Glasgow, Glasgow, Scotland; 2 Renal Department, NHS Grampian, Aberdeen, Scotland; 3 Population Health and Genomics, School of Medicine, University of Dundee, Dundee, Scotland; 4 General Practice and Primary Care, School of Health and Wellbeing, University of Glasgow, Glasgow, Scotland; 5 Renal and Transplant Unit, Queen Elizabeth University Hospital, Glasgow, Scotland

**Keywords:** Prognosis, Chronic renal failure, CKD, Multimorbidity, Frailty, Guidelines, Systematic review

## Abstract

**Background:**

Prognostic models that identify individuals with chronic kidney disease (CKD) at greatest risk of developing kidney failure help clinicians to make decisions and deliver precision medicine. It is recognised that people with CKD usually have multiple long-term health conditions (multimorbidity) and often experience frailty. We undertook a systematic review to evaluate the representation and consideration of multimorbidity and frailty within CKD cohorts used to develop and/or validate prognostic models assessing the risk of kidney failure.

**Methods:**

We identified studies that described derivation, validation or update of kidney failure prognostic models in MEDLINE, CINAHL Plus and the Cochrane Library—CENTRAL. The primary outcome was representation of multimorbidity or frailty. The secondary outcome was predictive accuracy of identified models in relation to presence of multimorbidity or frailty.

**Results:**

Ninety-seven studies reporting 121 different kidney failure prognostic models were identified. Two studies reported prevalence of multimorbidity and a single study reported prevalence of frailty. The rates of specific comorbidities were reported in a greater proportion of studies: 67.0% reported baseline data on diabetes, 54.6% reported hypertension and 39.2% reported cardiovascular disease. No studies included frailty in model development, and only one study considered multimorbidity as a predictor variable. No studies assessed model performance in populations in relation to multimorbidity. A single study assessed associations between frailty and the risks of kidney failure and death.

**Conclusions:**

There is a paucity of kidney failure risk prediction models that consider the impact of multimorbidity and/or frailty, resulting in a lack of clear evidence-based practice for multimorbid or frail individuals. These knowledge gaps should be explored to help clinicians know whether these models can be used for CKD patients who experience multimorbidity and/or frailty.

**Systematic review registration:**

This review has been registered on PROSPERO (CRD42022347295).

**Supplementary Information:**

The online version contains supplementary material available at 10.1186/s12916-024-03649-9.

## Key learning points

What was known:

- Individuals with CKD commonly experience multimorbidity and/or frailty.
- Multimorbidity and frailty are associated with adverse outcomes in CKD including disease progression.
- Clinical guidance recommends the use of validated risk prediction equations to guide referral to specialist kidney services, but it is unclear to what extent current prediction models have been developed with representative numbers of people with multimorbidity or frailty or if these syndromes impact model performance.

This study adds:

- Very few studies on kidney failure prognostic models reported multimorbidity or frailty prevalence or considered either as predictors.
- Kidney failure prognostic models have not been validated in relation to multimorbidity or frailty.

Potential impact:

- Clinical guidelines currently lack evidence for individuals with multimorbidity or frailty.
- Further research is required to support clinicians to know whether prognostic models recommended in clinical guidance can be used for individuals with CKD who experience multimorbidity and/or frailty.
- Validation of these models in sub-groups with multimorbidity and/or frailty will influence care for this population.

## Background

Chronic kidney disease (CKD) is increasing in prevalence, with current estimates reporting 10–15% of adults having a diagnosis of CKD [1]. Progression of CKD leads to kidney failure in a small proportion of people. Various factors influence the risk of kidney failure such as age of CKD onset, sex, ethnicity, lifestyle, underlying disease aetiology, blood pressure and glycaemic control [2]. Additionally, non-renal comorbidities that are worsened by or occur as a sequelae of kidney disease may also influence the progression of kidney disease [3, 4]. Identification of individuals at greatest risk of progressing to kidney failure requiring kidney replacement therapy (KRT), frequently referred to as end-stage kidney disease (ESKD), can be accomplished in risk prediction models. The use of such risk prediction models in clinical practice potentially provides more precise and individualised care. Application of prognostic models allows more informed patient communication, facilitates shared decision-making and guides evidence-based practice including timing of referral to specialist care and decisions around KRT planning.

Individuals with CKD are more likely to have increased numbers of long-term conditions compared to those without CKD [5]. Multimorbidity, the co-existence of two or more long-term conditions [6] differs conceptually from comorbidities where the focus is on a primary disease alongside co-existing conditions [7]. Both multimorbidity [8] and CKD [9, 10] are associated with experiencing frailty. Frailty can be described as a vulnerability to decompensation and impaired or delayed resolution of homeostasis in response to stressors, such as illness or medications [8, 11]. Frailty is also associated with advancing CKD, increased risk of hospitalisation and mortality [12]. There is an overlap between multimorbidity and frailty, but they are distinct concepts. Some individuals experience co-occurrence of multimorbidity and frailty, whilst others experience one but not the other [13]. There are a number of ways of measuring multimorbidity including weighted scores such as the Charlson [14] and Elixhauser [15] Comorbidity indices or simpler long-term condition counts [6]. Likewise, frailty can be measured in numerous ways with validated measures including but not limited to using cumulative deficit scores such as the frailty index [16, 17], the Fried frailty phenotype [18] and the clinical frailty scale [19] based on clinical evaluation of an individual.

Patients with CKD and multimorbidity are at increased risk of mortality [20] and hospitalisation [21] compared to CKD without multimorbidity. Multimorbidity has been shown to be associated with progression of CKD [20] and recent research highlights a cumulative risk of major adverse kidney events (KRT, doubling of creatinine, estimated glomerular filtration rate (eGFR) < 15 ml/min or > 30% decline in eGFR) with increasing numbers of long-term conditions [22].

Multiple risk prediction models have been developed to identify individuals at high risk of ESKD, with associated systematic reviews assessing their utility [23]. The Kidney Disease: Improving Global Outcome (KDIGO) 2013 guidance recommended the use of risk prediction models to guide referral for KRT planning and 2021 National Institute for Health and Care Excellence (NICE) guidelines for management of CKD [24] recommended the four variable kidney failure risk equation (KFRE) [25] to inform adults with CKD about their 5-year risk of need for KRT and to guide referral for specialist assessment. It is unclear to what extent current prediction models, including KFRE, have been developed with representative numbers of people with multimorbidity or frailty. Given these groups are at heightened risk of mortality, it is also unclear whether the competing risk of death has been appropriately factored into prediction tools. Heterogeneity between studies due to variation in study design, measurement of variables and outcomes and differences in case-mix may impact model performance [26].

The systematic review aimed to describe, summarise and evaluate the representation of multimorbidity and frailty within cohorts used to develop, validate and/or update prediction models assessing the risk of kidney failure in individuals with CKD over a minimum of 2 years.

Key objectives are as follows:

1. Evaluate if prevalence of multimorbidity or frailty are reported in studies that develop and/or validate kidney failure prediction models.
2. Assess the impact of multimorbidity and/or frailty, if any, that has been studied on the validity of models in estimation of kidney failure risk.

## Methods

The protocol for this systematic review was prospectively registered with the International Prospective Register of Systematic Reviews (PROSPERO) (registration number: CRD42022347295) and was prepared using the Preferred Reporting Items for Systematic Reviews and Meta-Analysis (PRISMA-P) 2015 statement [27] and the Critical Appraisal and Data Extraction for Systematic Reviews of Prediction Modelling Studies (CHARMS) checklist [28].

### Eligibility criteria

Articles eligible for inclusion were quantitative studies that developed, validated or updated a prognostic model for kidney failure, with the final model including a minimum of three predictors. The study population was adult patients with CKD, with a study outcome of kidney failure, ESKD or 50% reduction in baseline eGFR. Prediction models had to present at least one measure of model performance (calibration or discrimination).

Full inclusion and exclusion criteria are described in Additional file 1: Table S1-2.

### Information sources and search strategy

An electronic search for published peer-reviewed articles was applied to MEDLINE (EBSCO interface), CINAHL Plus (EBSCO interface) and the Cochrane Library—CENTRAL (OVID interface). Supplemented by manual review of reference lists of included studies and relevant clinical guidelines. Literature published up to 18 October 2023 was included. Full search strategies are presented in Additional file 1: Tables S3-5.

### Data management

Studies identified were imported into Rayyan systematic review software (https://www.rayyan.ai/). Covidence systematic review software (Covidence, Melbourne, Australia) was used for full text review, data extraction and risk of bias assessment.

### Data collection and analysis

Two reviewers (HW and SD) independently screened article titles and abstracts. Selected studies were reviewed independently, in full, to confirm eligibility. Differences were resolved by discussion with a third reviewer (PBM).

Data were extracted independently by HW and a second reviewer (SD, CHG, CJ or RK) into a pre-defined electronic data collection form including: study characteristics (author, publication year, country of origin, study design, start/end date, sample size), patient characteristics (including age, sex, ethnicity, eGFR, co-morbidities, multimorbidity/frailty measures), number of participants, number of events, outcomes (definitions, measurement, timing of measurement, duration of follow-up), model development (method for selection of predictors, model type, model variables) and model performance (calibration, discrimination measures). Where measures were not reported, we followed previously described methods to calculate missing performance measures where possible [26].

### Risk of bias

Two authors, HW and a second reviewer (SD, CHG, CJ or RK), independently reviewed the risk of bias using the Prediction model Risk Of Bias ASsessment Tool (PROBAST) [29]. PROBAST is a risk of bias assessment tool that has been developed through a consensus process specifically for assessment of risk of bias in systematic reviews of prediction model studies [29]. Differences were resolved by consensus with a third reviewer (PBM).

### Summary measures and data synthesis

Baseline characteristics are summarised using means with standard deviations, medians with interquartile ranges and frequencies with percentages as appropriate. Included studies and overall findings were reported using narrative synthesis. Where possible, data are presented in summary tables and graphical representation. Analyses were performed using R software version 4.3.0.

## Results

**Fig. 1:**
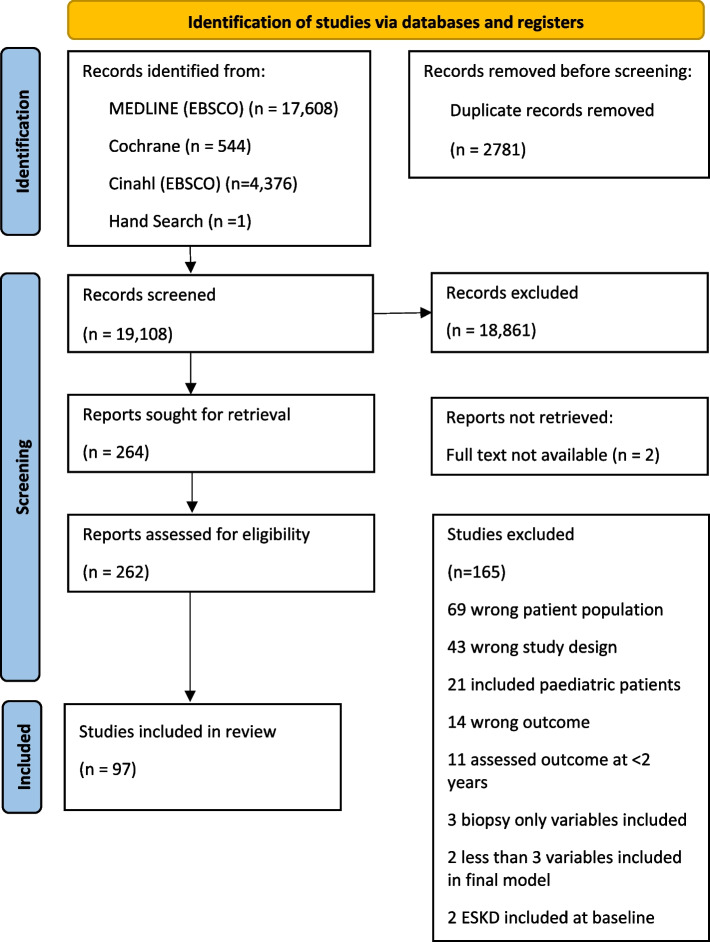
Flow chart of study selection

We identified 19,108 original articles, with 97 studies (full details of included studies in supplementary materials) [2, 25, 30–124] meeting the inclusion criteria, and a resultant 121 original kidney failure prognostic models. Overall, there were 2,925,413 participants and 149,380 kidney failure outcome events. Included participants had mean age of 58.9 years (SD 9.6), 44.4% were female, with mean eGFR of 47.5 ml/min/1.73m^2^ (SD 10.7). Figure 1 details the study selection process.

CKD definitions/sub-groups, populations and setting varied across included studies (Additional file 1: Table S6). Likewise, kidney failure outcome definitions and ascertainment (e.g. health records, laboratory data, coding or registry data) were not consistent.

Summaries of original models [125–134] and baseline characteristics of included studies are detailed in Additional file 1: Tables S7-8.

### Development studies

Fifty-four studies described the derivation of kidney failure prognostic models for use in CKD. There were a small number of prognostic models that were validated in studies included in the review, where the original development study did not meet inclusion criteria [125–134].

Thirty-one studies developed models for use in a general CKD population, although there was a wide variation in CKD definitions and eGFR cut-offs used across studies (Additional file 1: Table S6). The remaining included studies that developed/validated models to predict kidney failure in diabetic kidney disease (*n* = 15), IgA nephropathy (IgAN, *n* = 16), glomerular diseases (*n* = 2), focal segmental glomerulosclerosis (*n* = 1), ANCA-associated vasculitis (*n* = 1) and lupus nephritis (*n* = 1).

### Multimorbidity and frailty within included studies

The majority of studies included did not report the overall multimorbidity or frailty of participants. Only two studies reported multimorbidity (reported by Elixhauser Comorbidity Index (ECI) [95] and Charlson Comorbidity Index (CCI) [97]) and one study reported a frailty measure (self-reported general health scores as a proxy for frailty) [57]. Two additional studies reported a number of hospitalisations [37] and medications along with annual hospital admissions [53] which could be considered a proxy for multimorbidity or frailty. The study that reported ECI, was a prognostic model for CKD stages 3–4 and type 2 diabetes mellitus. Mean ECI was 4.6 (SD2.6), mean index in individuals who experienced kidney failure compared to those who did not was higher (6.1 (SD2.9) vs 4.8 (SD2.6), respectively). The ECI was considered for inclusion in the model but was not selected. However, ECI overlaps with individual comorbid conditions which form the index score, some of which were included in the model.

A single study that externally validated the 4-var KFRE in individuals with glomerulonephritides reported baseline CCI [97] with a median score of 2 (IQR 1–3). Discrimination was adequate, AUC 0.77 (95%CI 0.72–0.83) [97] but calibration was not reported. Table 1 and Additional file 1: Table S9 summarise the prognostic models and reporting of multimorbidity/frailty measures.

**Table 1:** Summary of kidney failure risk prediction models and multimorbidity/frailty measures reported for general CKD population

CKD population	Prediction models		Number of variables in the model	Number of external validation studies	Multimorbidity/frailty measure reported			
General CKD population	Johnson 2008		< 10	1				
	Landray			2	Prouvot 2021 – Octogenerian cohort but no frailty measure reported			
	Marks*							
	Schroeder KPNW score							
	Tangri	KFRE-3		3				
		KFRE-4		36	Hallan 2019—Self-reported general health score	Prouvot 2021 – Octogenerian cohort but no frailty measure reported	Stefan 2020—Charlson co-morbidity index	Bellocchio 2021 – number of hospitalisations
		KFRE-6		5				
		KFRE-8		11				
	Belur MLMs × 4			0				
	Dimitrov Renal Risk Index							
	Gibertoni CT-PIRP				Number of medications and annual hospital admissions as a proxy for disease severity			
	Hasegawa							
	Johnson 2007							
	Lim models 1–5							
	Smith							
	Tangri Dynamic model							
	Xu							
	Zacharias KFRT risk model							
	Zhu dynamic mode							
	Lee CKD stage 5 model							
	Maziarz model 1–2							
	Maziarz2 models 1–2							
	Xie model 1							
	Zhang models A-C							
	Led CKD stage 3 mode, CKD stage 4 model		10–20					
	Maziarz models 3–4							
	Maziarz2 models 3–4							
	Xie models 2–3							
	Zhang model D							
	Al-Wahsh							
	Dai							
	Edmonston, Endomonston + FGF23							
	Hsu base model, base model + urine biomarkers							
	Orlandi models 1–4							
	Sud							
	Yuan CKD3A model, CKD3B model							
	Grams Markov model		> 20	3	Prouvot 2021 – Octogenerian cohort but no frailty measure reported			
	Bellocchio PROGRESS-CKD			0	Number of hospitalisations			
	Bai 5 × MLMs		Unclear					

No studies assessed the performance or validation of kidney failure prognostic models in populations specifically with CKD and multimorbidity. The single study that used a self-reported general health measure as a proxy for frailty assessed the clinical utility of a model and referral algorithms considering frailty, risk of kidney failure and risk of death but did not assess discrimination or calibration measures in the context of frailty [57].

76.3% (*n* = 74) studies reported baseline data on one or more co-morbidities, primarily diabetes (67.0%, *n* = 65), hypertension (54.6%, *n* = 53) and cardiovascular disease (CVD) (39.2%, *n* = 38). Sixty-five studies including 1,610,586 participants reported on diabetes with a prevalence of 48.8%. A total of 1,565,364 participants from 54 studies reported on baseline hypertension with a pooled prevalence of 67.0%. Baseline CVD was reported in 39 studies, including 575,011 participants with a prevalence of 31.8%; however, there was heterogeneity in the definitions of CVD used across studies.

Forty-four studies considered the inclusion of one or more individual comorbidities as predictor variables in model development. Thirty-eight models went on to include one or more comorbidities as a variable in the reported models. Comorbidities included were most commonly diabetes (*n* = 28), hypertension (*n* = 19) and CVD (*n* = 18) but others also included chronic pulmonary disease, connective tissue disease, dementia, liver disease, anaemia, hyperlipidaemia, chronic viral disease and alcohol/drug misuse.

Table 2 and Additional file 2: Fig. S1 summarise reporting and inclusion of individual comorbidities for all models that have been independently externally validated.

**Table 2:**
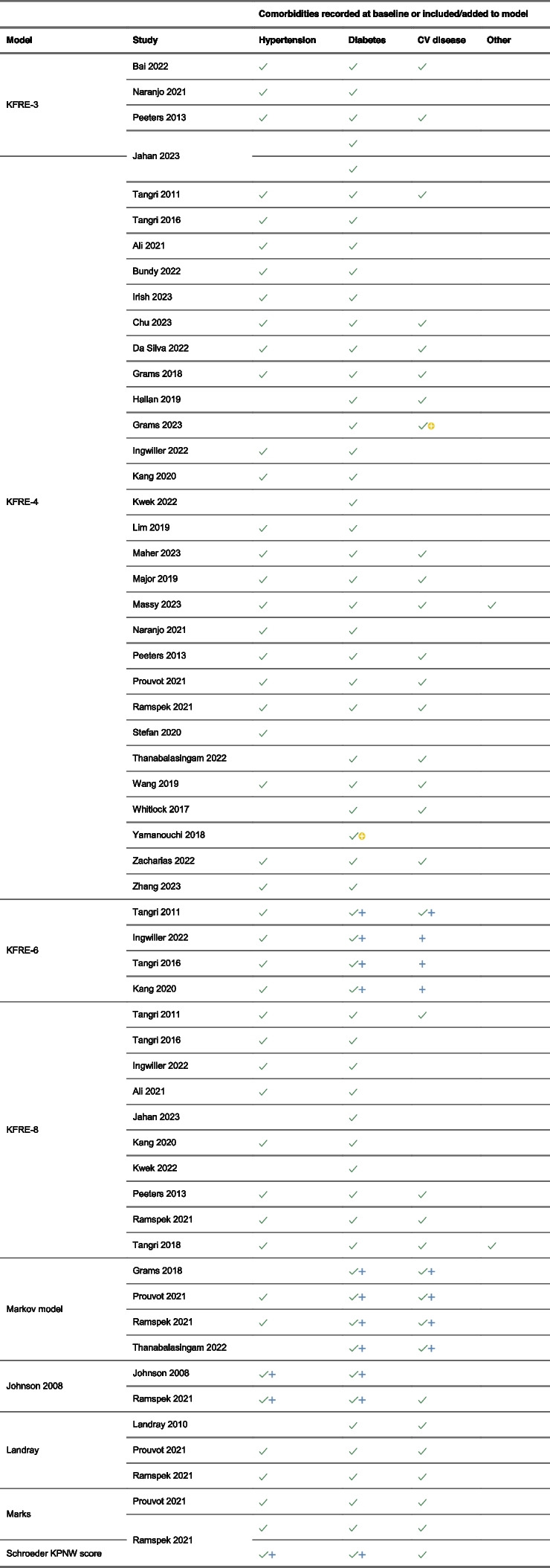
Baseline comorbidities recorded and included/added to externally validated kidney failure prediction models for general CKD population

*Original study developing model not included in the review

Model performance and observed and expected event numbers were not reported for the sub-groups of comorbidities in any study. Therefore, no meta-analysis of model performance by comorbidities was possible. Due to the number of included studies reporting on multimorbidity or frailty, no meta-analysis was possible.

### Kidney failure risk equation

The prognostic model that was most extensively validated was the four-variable KFRE (4-var KFRE) (*n* = 36). The 4-var KFRE performed well across most CKD populations and disease-specific aetiologies, discrimination (AUC or c-index) ranged from 0.59 to 0.98 at 2 years, 0.78–0.91 at 3 years and 0.57–0.96 at 5 years, with some recalibration factors applied in different regions/countries.

Although most studies that validated KFRE did not report on multimorbidity, 75.7% (*n* = 28) reported on baseline diabetes with a mean prevalence of 42.9%. Only 48.6% (*n* = 18) reported on hypertension and 43.2% (*n* = 16) on CVD with baseline prevalence reported at 77.9% and 34.4%, respectively.

One study [54] assessed the addition of previous CVD history into KFRE. Heart failure had a statistically significant association with kidney failure risk but did not improve overall model performance. A single study also assessed KFRE performance in the sub-groups by diabetes status and by those over 75 years compared to those 40–75 years old [106]. Discrimination was similar across different age groups but was lower in those with type two diabetes mellitus compared to those without. Likewise, the performance of 4-var KFRE has been explored by renal disease aetiology [61]. This study found that discrimination was adequate to excellent across all renal disease aetiologies explored but that calibration showed overestimated across all disease aetiologies with the exception of autosomal dominant polycystic kidney disease, where there was underestimation of kidney failure risk.

A further study [57] described validation of 4-var KFRE alongside the mortality risk equation for kidney disease (MREK) in patients over 65 years old although was limited by restricting definition of ESKD to those receiving KRT and not conservative care of advanced CKD.

The 4-var KFRE was also the model most frequently updated, with effectiveness of updating the model depending on variables added, with small or no improvement in model performance reported.

### Assessment of heterogeneity

Quantitative assessment of heterogeneity was not performed due to the inability to perform meta-analysis on included models.

### Risk of bias and applicability assessment

**Fig. 2:**
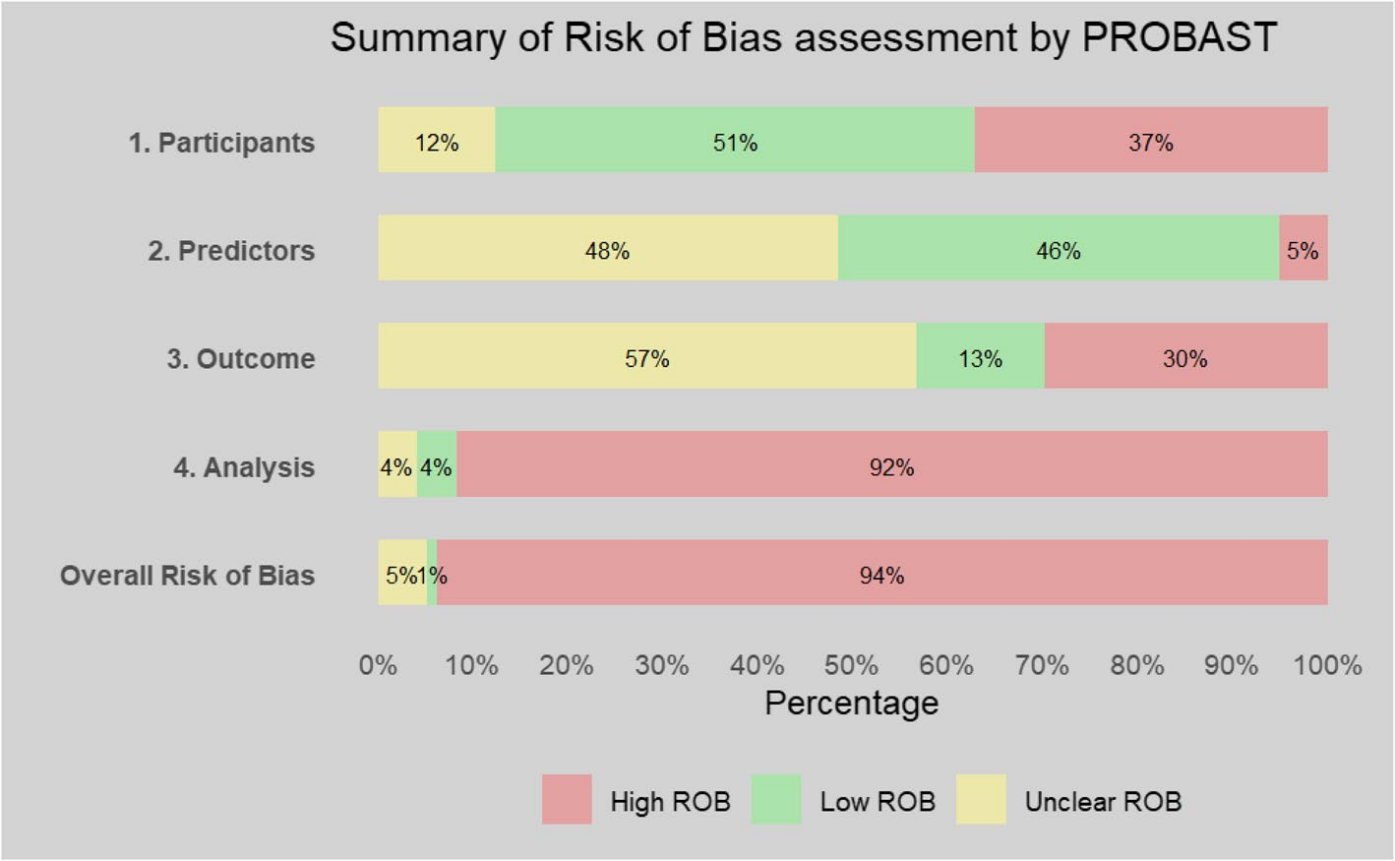
Summary of risk of bias assessment

**Fig. 3:**
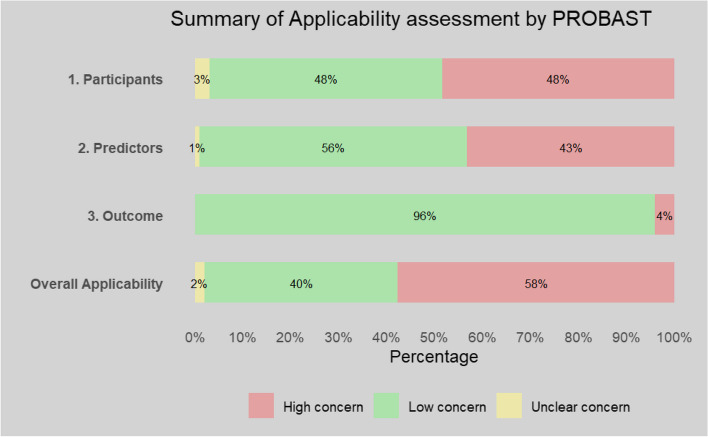
Summary of applicability assessment

Risk of bias was high, with 94% of studies having an overall high risk of bias. Summary of the PROBAST tool assessment of individual studies is presented in Additional file 1: Table S10 and overall and domain summaries for risk of bias and applicability assessment in Figs. 2 and 3.

High risk of bias was attributable mainly to lack of assessment or consideration of the competing risk of death (68 of 97 studies, Additional file 1: Table S11). Inclusion bias occurred in 30% of studies, where eGFR was included in both outcome definition and prognostic model. Only 21 of the original models provided the full model formula to allow replication or application. Forty-three studies were classed as high risk of bias due to not reporting both discrimination and calibration measures or only reporting goodness-of-fit tests, such as Hosmer–Lemeshow test, for calibration. Only 70% of studies reporting calibration (*n* = 50) did so graphically, which is the preferred form of reporting calibration [28].

There was a high concern regarding the applicability of 58% of the total included studies; this related to models focusing on the prediction of kidney failure in specific disease aetiologies of CKD such as IgA nephropathy rather than the general CKD population (48%) and the inclusion of model variables that were not routinely available in clinical practice (43%).

## Discussion

Prognostic models for the prediction of kidney failure infrequently report or include measures of multimorbidity or frailty. Due to the large variety of relevant prognostic models and the lack of reporting of multimorbidity or frailty, comparison of model performance was limited. This review has defined a gap in the research relating to consideration of the competing risk of death in prognostic models for kidney failure. This is important as traditional survival analyses, where data is left censored and assumes that individuals lost to follow-up have the same risk of the outcome as individuals who continue to be followed up. However, if an individual dies before the outcome occurs (competing mortality), then they cannot experience the outcome event (kidney failure). This may lead to an overprediction of kidney failure risk in individuals at higher risk of dying, such as those with multimorbidity or frailty. Competing mortality risk has been shown to impact predictive performance in other prognostic models and in older adults with more long-term conditions [135, 136]. The studies included in the review had a high risk of bias, primarily relating to lack of consideration of competing mortality risk and particular focus, and attention should be given to ensuring future studies employ appropriate methodologies and report studies in line with TRIPOD guidelines.

In keeping with a recent large scoping review of prognostic models for patients with CKD [137], including kidney failure prognostic models, our study found there was poor reporting of calibration. Calibration is important to consider alongside discrimination to assess overall model performance. There was also lack of reporting of full model formulas to allow replication or use of models. Similarly, the risk of bias findings in this review is consistent with a 2020 systematic review of prediction tools for kidney failure in CKD patients [23].

The two studies that reported a multimorbidity baseline measure used a weighted score, making them difficult to interpret. Therefore, it is not possible to determine from the information provided whether participants had multimorbidity based on the standard definition of the presence of two or more long-term conditions [6]. Within the studies that reported CCI and ECI, there is no comparison of model performance stratified by these measures.

Although baseline frailty or multimorbidity was not commonly reported in the existing prediction models, some studies were still relevant to these issues. Prouvout et al. [90] evaluated the performance of four kidney failure prognostic models [25, 55, 74, 132] in a cohort of octogenarian patients with advanced CKD. They found performance was poor across all models and highlighted that frailty may play a part in the poor performance of the prognostic models in this cohort. A 2016 systematic review of CKD and frailty [12] identified frailty as being associated with reducing eGFR and increased risk of adverse outcomes.

The inclusion of frailty may be limited by a lack of consensus on the optimal frailty measure to use in clinical practice or research settings. It is likely that frailty is not routinely available as a measure in observational data that are commonly used for the derivation or validation of prognostic models such as these. Frailty also fluctuates over time, with individuals moving between frailty states dependent on stressors such as illness or side effects from treatments. This may subsequently make it difficult to measure the true impact on subsequent kidney failure due to only capturing or accounting for an individual’s frailty status at a single point in time.

Although the most frequently validated prognostic model, the 4-var KFRE, performed well in general CKD populations and aetiology-specific CKD cohorts, there is evident heterogeneity between the populations and settings in which the model is validated. The performance of KFRE was reported as being lower in studies that focused on cohorts with glomerular diseases [120] and diabetic kidney disease [112]. The study by Maher et al. [79] highlighted that incorporating ethnicity and its interactions with key variables like urine albumin creatinine ratio (uACR) and eGFR significantly improved KFRE’s performance, for South Asian populations. Likewise, deficiencies in KFRE performance were noted by Grams et al. (2023) [54] with underprediction of kidney failure in the subgroup of individuals ≥ 65 years of age and overestimation of risk at 5-year timeframes. Individuals are more likely to have increasing levels of multimorbidity and frailty as age increases and should be considered as factors that could impact model performance. Additionally, some studies that have considered the competing risk of death in KFRE have highlighted improvement in performance [54, 79]. Of particular interest is the finding that competing risk analysis improved calibration in the subgroups of older adults (≥ 65 years old) and those with eGFR between 45 and 59 ml/min/1.73m^2^ [54].

A number of studies considered one or more individual comorbidities as predictor variables in model derivation, with the majority going on to include at least one comorbidity as a variable in the model developed. Comparison of these studies is limited by the variation in methodologies used to decide on predictor variable inclusion. Increasing numbers of machine learning models are being used to develop prognostic models for use in healthcare settings. Such methodologies allow consideration of a greater number of variables and exploration of more complex relationships than more traditional statistical methods. One such excluded study (no reported performance measures) developed a model using natural language processing to identify disease clusters based on ICD-9 codes, with improvements in classification accuracy using a machine learning model compared to more traditional models that included comorbidities.

This systematic review has several strengths, including its extensive search strategy supporting the identification and comprehensive overview of available kidney failure prognostic models available for general and disease aetiology-specific CKD populations. In addition, all review stages were performed in duplicate and independently adding to the robustness of study identification and data extraction. A further strength is the formal risk of bias and applicability assessment conducted using PROBAST. However, the overall finding of high risk of bias is not a novel one with a meta-review of prediction model systematic review literature identifying high risk of bias in the majority of studies and over time that this predominantly applied to the analysis domain [138], as we have found in this study. This, however, highlights the ongoing need for prediction model studies to focus on improving methodological and model quality. The review is also limited by not all kidney failure prognostic model studies being included due to the lack of reporting of performance measures. Additionally, we were unable to perform meta-analysis due to the small number of studies reporting on multimorbidity or frailty.

This study has identified a gap in the research for the use of kidney failure prognostic models in adults with CKD and multimorbidity or frailty including how the presence of these factors influences model performance. Additionally, understanding and comparison of model performance and use is limited by heterogeneity in CKD and kidney failure outcome definitions used and by differences in the timing of outcome assessment.

NICE and KDIGO guidelines both promote the use of prognostic models to predict the risk of kidney failure to guide the referral and treatment of patients with CKD. Validation of KFRE has been produced in multinational cohorts, varied settings and in different disease aetiologies but not specifically with a focus on multimorbidity or frailty. Given there is a high prevalence of multimorbidity and frailty in individuals with CKD, it is essential that multimorbidity and frailty are represented within the cohorts used to develop, validate, and update available models. There is currently a lack of reporting of multimorbidity or frailty measures to say with certainty that such models are valid for use in individuals with multimorbidity or frailty.

Future research should focus on the assessment of validated prediction models that are recommended in clinical guidelines in those with multimorbidity or frailty compared to those without. Existing models may benefit from recalibration, updating or consideration of competing risks to improve performance to take account of multimorbidity and/or frailty.

## Conclusions

Multimorbidity and frailty are not yet well understood. In this review, we have been unable to determine how available prognostic models for kidney failure perform in individuals with multimorbidity and/or frailty. Further research is required to ensure that kidney failure prognostic and prediction models have sufficient clinical utility for use in these patients and should potentially be optimised to take account of these conditions where appropriate.

## Supplementary Information

Additional file 1: Tables S1–S11. Table S1. Full inclusion criteria. Table S2. Full exclusion criteria. Table S3. Medline (EBSCO interface) search strategy. Table S4. CINHAL (EBSCO interface) search strategy. Table S5. Cochrane Library – CENTRAL search strategy. Table S6. Overview of included studies – population, setting CKD definitions, outcome definitions, eGFR equations used. Table S7. Summary of original models. Table S8. Baseline characteristics of included studies. Table S9. Summary of kidney failure risk prediction models and multimorbidity/frailty measures reported for renal specific disease aetiologies. Table S10. Summary of PROBAST tool assessment of individual studies. Table S11. Competing risk of death consideration and performance measures of included.

Additional file 2: Figure S1. Baseline co-morbidities recorded and included/added to externally validated kidney failure prediction models for renal specific disease aetiologies.

## Data Availability

All data generated of analysed during this study are included in this published article and its supplementary information files.

## Abbreviations

CKD: Chronic kidney disease
KRT: Kidney replacement therapy
ESKD: End-stage kidney disease
eGFR: Estimate glomerular filtration rate
uACR: Urine albumin-to-creatinine ratio
KDIGO: Kidney Disease: Improving Global Outcomes
NICE: National Institute for Health Care Excellence
KFRE: Kidney failure risk equation
PROSPERO: The International Prospective Register of Systematic Reviews
PRISMA-P: Preferred Reporting Items for Systematic Reviews and Meta-Analysis Protocols
CHARMS: Checklist for critical appraisal and data extractions for systematic reviews of prediction modelling studies
PROBAST: Prediction model risk of bias assessment tool
IgAN: IgA nephropathy
ECI: Elixhauser comorbidity index
CCI: Charlson Comorbidity Index
AUC: Area under the receiver operating characteristic curve
CVD: Cardiovascular disease
4-var KFRE: Four-variable kidney failure risk equation
MEST: Mesangial hypercellularity (M), endocapillary hypercellularity (E), segmental glomerulosclerosis (S), tubular atrophy/interstitial fibrosis (T)
AAV: ANCA-associated vasculitis
MLM: Machine learning model
DKD: Diabetic kidney disease
T2DM: Type two diabetes mellitus
Cox PH model: Cox proportional-hazards model
MDT: Multi-disciplinary team
MAP: Mean arterial pressure
RASB: Renin-angiotensin system blockade
PTH: Parathyroid hormone
CHF: Congestive heart failure
CTD: Connective tissues disease
CAD: Coronary artery disease
SBP: Systolic blood pressure
RBC: Red blood cell
NLR: Neutrophil to lymphocyte ratio
PVD: Peripheral vascular disease
CRP: C-reactive protein
AKI: Acute kidney injury
TnT: Troponin T
BNP: Brain natriuretic peptide
UPE: Urine protein excretion
FGF23: Fibroblast growth factor 23
HbA1C: Glycated haemoglobin
TNFalphaR1: Tumour necrosis factor-α receptor 1
GN: Glomerulonephritis
PKD: Polycystic kidney disease
NT–pro-BNP: N-Terminal pro-B-type natriuretic peptide
DBP: Diastolic blood pressure
COPD: Chronic obstructive pulmonary disease
uPCR: Urine protein-to-creatinine ratio
uNCR: Urinary neutrophil gelatinase-associated lipocalin-to-creatinine ratio
uMMP7: Urinary matrix metalloproteinase 7
AGT: Angiotensinogen
EGF: Epidermal growth factor
K1M1: Kidney injury molecule 1
Gd-IgA1: Galactose-deficient immunoglobulin A1
ALT: Alanine aminotransferase test
HDL: High density lipoprotein
LDL: Low density lipoprotein
MCV: Mean cell volume
ISN/RPS: International Society of Nephrology and the Renal Pathology Society
MST: Mesangial hypercellularity (M), segmental glomerulosclerosis (S), tubular atrophy/interstitial fibrosis (T)
O:E: Observed versus predicted
