# Supplementary material for: Representation of multimorbidity and frailty in the development and validation of kidney failure prognostic prediction models: a systematic review
Source: BMC Med. 2024 Oct 11;22:452. doi: 10.1186/s12916-024-03649-9 (PMC11470573; doi:10.1186/s12916-024-03649-9)
Supplement: Supplementary file 1 — Additional file 1: Tables S1–S11. Table S1. Full inclusion criteria. Table S2. Full exclusion criteria. Table S3. Medline (EBSCO interface) search strategy. Table S4. CINHAL (EBSCO interface) search strategy. Table S5. Cochrane Library – CENTRAL search strategy. Table S6. Overview of included studies – population, setting CKD definitions, outcome definitions, eGFR equations used. Table S7. Summary of original models. Table S8. Baseline characteristics of included studies. Table S9. Summary of kidney failure risk prediction models and multimorbidity/frailty measures reported for renal specific disease aetiologies. Table S10. Summary of PROBAST tool assessment of individual studies. Table S11. Competing risk of death consideration and performance measures of included. [file 12916_2024_3649_MOESM1_ESM.docx]

**Additional file 1**

**Additional file 1: Table S1. Full inclusion criteria**

| Inclusion Criteria | Definition |
| --- | --- |
| Population | Adult patients aged 18 years or older with CKD (definition as per individual studies) with no previous history of kidney failure or pre-emptive kidney transplantation. |
| Study Design | Articles reporting the development and/or validation or update of a prediction model with at least one measure to assess model performance. |
| Year of publication | From inception to 18^th^ October 2023 |
| Minimum of 3 predictors in model | Articles reporting the development and/or validation or update of a prediction model using a minimum of three predictor variables. At least one of these predictors should be a clinical or demographic variable. |
| Outcome | Kidney failure/ESKD (as per individual study definitions) or 50% reduction in baseline eGFR. |
| Time-to-event | Minimum of 2 years. |

**Additional file 1: Table S2. Full exclusion criteria**

| Exclusion Criteria | Definition |
| --- | --- |
| Population | Adult patients aged less than 18 years old, the general population or groups without CKD and individuals with a history of kidney failure or kidney transplantation. |
| Study Design | Articles reporting drug intervention studies, qualitative studies, narrative or systematic reviews.  Articles that report the identification of prognostic factors associated with the outcome. |
| Predictors in model | Articles reporting prediction models using biopsy/pathology or genetic predictors only. |
| Full text not available | Articles for which the full text is not accessible online or through accessible library resources. |
| Language | Articles not available in English language. |

**Additional file 1: Table S3.**

**Medline (EBSCO interface) search strategy**

| #1 | (MH "Risk Assessment/MT") |
| --- | --- |
| #2 | (MH "Prognosis") |
| #3 | (MH "Disease Progression") |
| #4 | AB ( risk n3 (predict* OR model* OR progress* OR scor* OR factor* OR tool* OR equation*) ) OR TI ( risk n3 (predict* OR model* OR progress* OR scor* OR factor* OR tool* OR equation*) ) |
| #5 | AB ( predict* n4 (model* OR progress* OR scor* OR factor* OR tool* OR disease* OR equation*) ) OR TI ( predict* n4 (model* OR progress* OR scor* OR factor* OR tool* OR disease* OR equation*) ) |
| #6 | S1 OR S2 OR S3 OR S4 OR S5 |
| #7 | (MH "Kidney Failure, Chronic/BL/CO/DI/EP/PP/TH/UR") |
| #8 | (MH "Renal Insufficiency, Chronic/BL/CO/EP/PP/UR/TH/DI") |
| #9 | (MH "Renal Replacement Therapy/MT/TD") |
| #10 | AB ( RRT OR KRT ) OR TI ( RRT OR KRT ) |
| #11 | AB ( (kidney OR renal) AND replacement* ) OR TI ( (kidney OR renal) AND replacement* ) |
| #12 | AB CKD OR TI CKD |
| #13 | AB "chronic kidney disease" OR TI "chronic kidney disease" |
| #14 | AB ( (kidney OR renal) AND failure ) OR TI ( (kidney OR renal) AND failure ) |
| #15 | AB ( (end-stage* OR endstage*) AND (renal OR kidney) ) OR TI ( (end-stage* OR endstage*) AND (renal OR kidney) ) |
| #16 | AB ( chronic AND (renal OR kidney) AND (insufficiency OR impairment) ) OR TI ( chronic AND (renal OR kidney) AND (insufficiency OR impairment) ) |
| #17 | AB ( ESRD OR ESKD ) OR TI ( ESRD OR ESKD ) |
| #18 | AB nephropath* OR TI nephropath* |
| #19 | S7 OR S8 OR S9 OR S10 OR S11 OR S12 OR S13 OR S14 OR S15 OR S16 OR S17 OR S18 |
| #20 | (MH "Humans") |
| #21 | (MH "Adult") |
| #22 | (MH "Middle Aged") |
| #23 | S20 OR S21 OR S22 |
| #24 | Develop* |
| #25 | Validat* |
| #26 | S24 OR S25 |
| #27 | S6 AND S19 AND S23 AND S26 |
|  | Limit to English language |

**Additional file 1: Table S4.**

**CINAHL (EBSCO interface) search strategy**

| #1 | (MH "Risk Assessment/MT") |
| --- | --- |
| #2 | (MH "Prognosis") |
| #3 | (MH "Disease Progression") |
| #4 | AB ( risk n3 (predict* OR model* OR progress* OR scor* OR factor* OR tool* OR equation*) ) OR TI ( risk n3 (predict* OR model* OR progress* OR scor* OR factor* OR tool* OR equation*) ) |
| #5 | AB ( predict* n4 (model* OR progress* OR scor* OR factor* OR tool* OR disease* OR equation*) ) OR TI ( predict* n4 (model* OR progress* OR scor* OR factor* OR tool* OR disease* OR equation*) ) |
| #6 | S1 OR S2 OR S3 OR S4 OR S5 |
| #7 | (MH "Kidney Failure, Chronic/BL/CO/DI/EP/PP/TH/UR") |
| #8 | (MH "Renal Insufficiency, Chronic/BL/CO/EP/PP/UR/TH/DI") |
| #9 | (MH "Renal Replacement Therapy/MT/TD") |
| #10 | AB ( RRT OR KRT ) OR TI ( RRT OR KRT ) |
| #11 | AB ( (kidney OR renal) AND replacement* ) OR TI ( (kidney OR renal) AND replacement* ) |
| #12 | AB CKD OR TI CKD |
| #13 | AB "chronic kidney disease" OR TI "chronic kidney disease" |
| #14 | AB ( (kidney OR renal) AND failure ) OR TI ( (kidney OR renal) AND failure ) |
| #15 | AB ( (end-stage* OR endstage*) AND (renal OR kidney) ) OR TI ( (end-stage* OR endstage*) AND (renal OR kidney) ) |
| #16 | AB ( chronic AND (renal OR kidney) AND (insufficiency OR impairment) ) OR TI ( chronic AND (renal OR kidney) AND (insufficiency OR impairment) ) |
| #17 | AB ( ESRD OR ESKD ) OR TI ( ESRD OR ESKD ) |
| #18 | AB nephropath* OR TI nephropath* |
| #19 | S7 OR S8 OR S9 OR S10 OR S11 OR S12 OR S13 OR S14 OR S15 OR S16 OR S17 OR S18 |
| #20 | (MH "Human") |
| #21 | (MH "Adult") |
| #22 | (MH "Middle Age") |
| #23 | S20 OR S21 OR S22 |
| #24 | Develop* |
| #25 | Validat* |
| #26 | S24 OR S25 |
|  | S6 AND S19 AND S23 AND S26 |
|  | Limit to English language |

**Additional file 1: Table S5.**

**Cochrane Library – CENTRAL search strategy**

| #1 | MeSH descriptor: [Risk Assessment] this term only |
| --- | --- |
| #2 | MeSH descriptor: [Prognosis] this term only |
| #3 | MeSH descriptor: [Disease Progression] this term only |
| #4 | (risk near/3 (predict* or model* or progress* or scor* or factor* or tool* or equation*)):ti,ab |
| #5 | (predict* near/4 (model* or progress* or scor* or factor* or tool* or disease* or equation*)):ti,ab |
| #6 | {or #1-#5} |
| #7 | MeSH descriptor: [Kidney Failure, Chronic] this term only |
| #8 | MeSH descriptor: [Renal Insufficiency, Chronic] this term only |
| #9 | MeSH descriptor: [Renal Replacement Therapy] this term only |
| #10 | (RRT or KRT):ti,ab |
| #11 | ((kidney or renal) and replacement*):ti,ab |
| #12 | CKD:ti,ab |
| #13 | chronic kidney disease:ti,ab |
| #14 | ((kidney or renal) and failure):ti,ab |
| #15 | ((end-stage* or endstage*) and (renal or kidney)):ti,ab |
| #16 | (chronic and (renal or kidney) and (insufficiency or impairment)):ti,ab |
| #17 | (ESRD or ESKD):ti,ab |
| #18 | nephropath*:ti,ab |
| #19 |  |
| #20 | MeSH descriptor: [Humans] this term only |
| #21 | MeSH descriptor: [Adult] this term only |
| #22 | MeSH descriptor: [Middle Aged] this term only |
| #23 | {or #20-#22} |
| #24 | Develop*:ti,ab |
| #25 | Validat*:ti,ab |
| #26 | #6 and #19 and #23 and #26 |

**Additional file 1: Table S6. Overview of included studies – population, setting, CKD definition, outcome definitions, eGFR equations used.**

| Author | Model | Year | Cohort | CKD definition | Setting | Outcome definition | eGFR equation used |
| --- | --- | --- | --- | --- | --- | --- | --- |
| Ali(29) | KFRE-4 KFRE-8 | 2021 | Patients referred to advanced kidney care service | eGFR<30 | Secondary care | KRT or conservative care | CKD-EPI |
| Al-Wahsh(28) | Al-Wahsh | 2021 | General Canadian population data with stage 4 CKD | eGFR 15- <30 | Primary & secondary care | KRT or eGFR<10 | CKD-EPI |
| Bai(30) | 5 MLMs  KFRE-3 | 2022 | Chinese CKD population | CKD stages 1-5 | Secondary care | KRT | Not reported |
| Bai(31) | Renal Risk Score | 2021 | Chinese AAV cohort | AAV on renal biopsy | Secondary care | KRT | CKD-EPI |
| Barbour(32) | IgA prediction tool  Updated IgA prediction tool | 2022 | European pts from VALIGA study, Chinese, Japanese + European/American pts from Oxford study | Biopsy proven IgAN | Secondary care | eGFR <15 mL/min/1.73m2, KRT or a permanent reduction in eGFR to <50% of the value at baseline | CKD-EPI |
| Barbour(33) | IgA prediction tool  Clinical model  Limited model | 2019 | Multi-ethnic cohort from VALIGA study, Oxford study, 2 Chinese & 1 Japanese cohorts | Biopsy proven IgAN | Secondary care | eGFR <15 mL/min/1.73m2, KRT or a permanent reduction in eGFR to <50% of the value at baseline | CKD-EPI |
| Barbour(34) | Clinical model  Clinical model + MEST score | 2016 | Pooled cohorts from VALIGA, Oxford + North American validation studies with IgAN | Biopsy proven IgAN | Secondary care | eGFR <15 mL/min/1.73m2 or a permanent reduction in eGFR to <50% of the value at baseline | MDRD |
| Bellocchio(35) | PROGRESS-CKD KFRE-4  KFRE-6 | 2021 | FMC Nephro-Care cohort (EuClid) + GCKD study cohort | eGFR<60 non-dialysis dependent | Secondary care | KRT | CKD-EPI 2009 |
| Belur(36) | 4 MLMs, Cox PH model  KFRE-8 | 2020 | Adult patients with DKD/T2DM from RENAAL, IDNT + ALTITUDE trials | T2DM + proteinuria (proteinuria levels set for each trial) | Clinical trial | KRT or doubling of serum creatinine from baseline | MDRD |
| Bon(37) | IgA prediction tool with and without ethnicity | 2023 | French biopsy cohort | Biopsy proven IgAN | Secondary care | KRT or sustained reduction in eGFR <50% of baseline value | CKD-EPI 2009 |
| S  dy(38) | KFRE-4 | 2022 | CRIC study participants | eGFR 20-70 | Study data | KRT | CKD-EPI (compares old & new equations and Cr, Cys + CrCys equations) |
| Chen(39) | Chen MLM + cox regression model | 2019 | Chinese IgAN cohort | Biopsy proven IgAN, eGFR ≥30 + proteinuria ≥0.5g/day | Secondary care | eGFR < 15 mL/min/1.73 m2 for more than 3 months or KRT or 50% reduction in eGFR | CKD-EPI |
| Cheng(40) | Cheng – clinical model  Lab model  Lab-medication model  Full model | 2020 | Chinese hospitalised patients with DKD | Biopsy proven DKD, eGFR 30-60 or albuminuria | Secondary care | KRT | CKD-EPI |
| Chu(41) | KFRE-4 | 2023 | CKD Outcomes and Practice Patterns Study (CKDOPPS) cohort | eGFR<60 | Secondary care | KRT | CKD-EPI |
| da Silva(42) | KFRE-4 | 2023 | CKD population referred for vascular access | eGFR<60 | Secondary care | KRT | CKD-EPI |
| da Silva(43) | KFRE-4 | 2022 | Patients referred to Portuguese nephrology clinic | eGFR<60 | Secondary care | KRT | CKD-EPI |
| Dai(44) | Dai | 2021 | CKD population identified from administrative claims database | CKD stage 3 or 4 (eGFR 15-60 or ICD-9/10 code) | Administrative claims database (Primary & secondary care) | ≥1 medical claim for stage 5 CKD (ICD-10-CM: N18.5) or ESRD (ICD-10-CM: N18.6); ≥1 medical claim for dialysis therapy or kidney transplantation; an eGFR <15 | MDRD |
| Desai(45) | TREAT ESRD model + cardiac biomarkers | 2011 | TREAT trial participants | eGFR 20-60 + T2DM + anaemia | Clinical trial | KRT (sustained for at least 30 days or death within 30 days), a physician recommendation to initiate dialysis therapy with documented patient refusal | MDRD |
| Dimitrov(46) | Renal Risk Index | 2003 | REIN study participants | CrCl 20-70 or >1g/24hrs proteinuria | Clinical trial data | Not described | measured GFR unlabelled iohexol |
| Edmonston(47) | Edmonston + FGF23 | 2019 | CRIC study participants | eGFR 20-70 | Secondary care study data | KRT | CRIC study equation |
| Fenton(48) | KFRE-4 + FLCs | 2018 | RIISC study participants with CKD | eGFR<30 or 30-59 with decline ≥5ml/min over 1 yr or ≥10ml over 5yrs or ACR ≥70mg/mmol | Secondary care study data | KRT | CKD-EPI |
| Floyd(49) | RRS (Brix) | 2023 | Multinational biopsy cohort | Biopsy proven Anti-GBM disease | Secondary care and registries | KRT | CKD-EPI |
| Forsblom(50) | Forsblom | 2014 | FinnDiane cohort study participants with T1DM + nephropathy | DKD - T1DM + macroalbuminuria (≥300mg/day or ≥200mcg/min) | Study data primary & secondary care | KRT | CKD-EPI |
| Gibertoni(51) | CT-PIRP | 2019 | PIRP project participants | Patients referred to nephrology centres by primary care physicians | Primary and secondary care | KRT | CKD-EPI |
| Grams(52) | Markov model KFRE-4 | 2018 | CKD prognosis consortium (CKD-PC) | eGFR <30 | Primary & secondary care | KRT | CKD-EPI 2009 |
| Grams(53) | KFRE-4 plus other variables | 2023 | CKD prognosis consortium (CKD-PC) | eGFR <60 | Primary & secondary care | KRT | CKD-EPI 2021 + 2009 |
| Haaskjold(54) | IgA prediction tool  IgA CDSS (Schena et al.) | 2023 | Norwegian Kidney Biopsy Registry | Biopsy proven IgAN, eGFR>30 | Biopsy Registry | KRT, eGFR<15ml/min/1.73m^2^ or decline in eGFR of 50% | Not reported |
| Hallan(55) | KFRE-4 | 2019 | HUNT study CKD 4 participants aged >65 years old | eGFR<45 | Cohort study data | KRT | CKD-EPI |
| Hasengawa(56) | Hasegawa | 2019 | CKD-Japan Cohort (CKD-JAC) | eGFR<60 | Secondary care | KRT | Japanese eGFR equation |
| Hoshino(57) | Hoshino | 2015 | Japanese biopsy cohort | Biopsy proven DKD | Secondary care | KRT | Unclear |
| Hsu(58) | Hsu | 2017 | CRIC study cohort | eGFR 20-70 | Study data secondary care | KRT or reduction in eGFR to <50% of the value at baseline | CRIC study equation |
| Hundemer(59) | KFRE-4 | 2020 | Canadian CKD patients referred to tertiary multi-care kidney clinic | Referred to Multi-Care Kidney clinic | Secondary care | KRT | CKD-EPI |
| Hwang(60) | Oxford model  IgA prediction tool | 2021 | Patients >20 yrs old with IgAN on biopsy | Biopsy proven IgAN | Secondary care | eGFR <15ml/min/1.73m^2^, KRT or reduction in eGFR to <50% of the value at baseline | CKD-EPI |
| Ingwiller(61) | KFRE-4 KFRE-6 KFRE-8 | 2022 | French CKD cohort | eGFR<60, non-dialysis | Secondary care | KRT | CKD-EPI |
| Irish(62) | KFRE-4 | 2023 | Australian, New Zealand and Tasmanian CKD cohort | eGFR<60 | Study data | KRT  Sensitivity analysis using eGFR<10 ml/min/1.73m^2^ and <7.5 ml/min/1.73m^2^ | CKD-EPI |
| Jahan(63) | KFRE-3, KFRE-4, KFRE-8 | 2023 | Patients under the care of an Australian CKD MDT programme | eGFR<60, non-dialysis dependent | Secondary care | KRT | Not reported |
| Jiang(64) | Jiang | 2019 | Chinese biopsy cohort | Biopsy proven DKD | Secondary care | KRT | CKD-EPI |
| Johnson(65) | Johnson 2008 | 2008 | Health maintenance organisation CKD cohort | eGFR 15-59 | Primary & secondary care | KRT | MDRD |
| Johnson(66) | Johnson 2007 | 2007 | Health maintenance organisation CKD cohort | eGFR<60 | Health maintenance organisation | KRT | MDRD |
| Kang(67) | KFRE-4, KFRE-6, KFRE-8 | 2020 | Adult patients with CKD3-5 Korean population | eGFR<60 | Secondary care | KRT | MDRD |
| Knoop(68) | ARR | 2015 | Norwegian kidney biopsy registry IgAN cohort | Biopsy proven IgAN | Secondary care registry | KRT | CKD-EPI |
| Kong(69) | RRS (Brix) | 2023 | Chinese biopsy cohort | Biopsy proven MPO AAV | Secondary care | KRT | Not reported |
| Kwan(70) | Kwan | 2020 | CRIC study participants with DKD | eGFR 20-70, diabetes + albuminuria | Study data secondary care | KRT | CRIC study equation, CKD-EPI |
| Kwek(71) | KFRE-4, KFRE-8 | 2022 | Multi-ethnic Singapore cohort referred to renal hospital department | eGFR<60 | Secondary care | KRT | CKD-EPI |
| Landray(72) | Landray | 2010 | CRIB study participants with CKD | eGFR<60 | Secondary care study data | KRT | MDRD |
| Lee(73) | Lee | 2018 | Sout Korean hospital cohort | eGFR<60ml | Secondary care | KRT | CKD-EPI, MDRD |
| Lennartz(74) | KFRE-4 + resistive index | 2016 | CARE FOR HOMe + Hannover cohort participants | CKD 2-4, CrCl <75% of their normal value for age and sex, proteinuria ≥150 mg/d or hypertension, or other established CKD | Secondary care | KRT or insertion of a peritoneal dialysis catheter | MDRD |
| Lim(75) | Lim  KFRE-4 | 2019 | Singapore Epidemiology of Eye Disease study participants with CKD | eGFR<60 or albuminuria >30mg/g | Secondary care study data | eGFR <15ml/min/1.73m^2^, serum creatinine >500 μmol/L or KRT | CKD-EPI |
| Lin(76) | Lin | 2023 | Action to Control Cardiovascular Risk in Diabetes (ACCORD) clinical trial | CKD + T2DM (not fully described) | Clinical trial | KRT or eGFR <15ml/min/1.73m^2^, | MDRD |
| Maher(77) | KFRE-4, KFRE-4 + ethnicity | 2023 | UK primary care cohort | eGFR<60 | Primary care | KRT | CKD-EPI, MDRD |
| Major(78) | KFRE-4 | 2019 | UK primary care cohort | eGFR<60 | Primary care | KRT | CKD-EPI, MDRD |
| Massy(79) | KFRE + Urine Peptidome | 2023 | French CKD-Renal Epidemiology and Information Network (REIN) cohort | eGFR<60 | Secondary care | KRT | CKD-EPI |
| Maziarz(80) | Maziarz | 2015 | American safety net healthcare systems, homeless cohort | eGFR<60 | Primary and secondary care Urban poor population | KRT | MDRD |
| Maziarz(81) | Maziarz2 | 2014 | American safety net healthcare systems | eGFR<60 | Primary and secondary care (homeless and poor population) | KRT | MDRD |
| Naranjo*82) | KFRE-3, KFRE-4 | 2021 | American electronic health records system | eGFR<60 | Secondary care outpatient | KRT or CKD stage 5 | CKD-EPI |
| Orlandi(83) | Orlandi | 2018 | CRIC study participants | eGFR 20-70 | Primary and secondary care study data | ≥50% decrease in eGFR from baseline or KRT | CRIC study equation |
| Ouyang(84) | Barbour IgA prediction tool 2019, KFRE-4 | 2021 | Multicentre Chinese biopsy cohort | Biopsy proven IgAN + eGFR>15 | Secondary care | 50% decline in eGFR, eGFR <15ml/min/1.73m^2^ or KRT | CKD-EPI |
| Park(85) | Hass classification, Oxford classification models | 2014 | Korean biopsy cohort | Biopsy proven IgA | Secondary care | doubling of the baseline serum creatinine concentration or KRT | CKD-EPI |
| Peeters(86) | KFRE-3, KFRE-4, KFRE-8 | 2013 | MASTERPLAN cohort | eGFR<60 | Clinical trial | KRT | MDRD |
| Pesce(87) | Pesce, Berthoux | 2016 | Multinational IgAN cohorts | Biopsy proven IgAN | Secondary care | KRT | CKD-EPI |
| Prouvot(88) | Grams, KFRE-4, Landray, Marks | 2021 | PSPA participants >75 years old | eGFR <20ml | Secondary care | KRT | MDRD |
| Ramspek(89) | Grams, KFRE-4, KFRE-8, Landray, Marks, KPNW score (Schroeder et al. 2016), Johnson 2008 | 2021 | EQUAL study participants ≥65 years old and SRR cohort | eGFR 8-30ml | Secondary care | KRT | CKD-EPI, MDRD |
| Sato(90) | Japanese histologic classification 2013 | 2015 | Japanese biopsy cohort | Biopsy proven IgAN | Secondary care | 50% decline in eGFR or KRT | Japanese eGFR equation |
| Schena(91) | Schena (CDSS - ANN model, Berthoux, Tanaka, Barbour models) | 2021 | VALIGA cohort study participants and Greek hospital biopsy cohort | Biopsy proven IgA | Secondary care | eGFR<15ml/min/1.73m^2^ or KRT | MDRD |
| Schroeder(92) | Schroeder | 2017 | American health maintenance organisation | eGFR 15-59 | Secondary care outpatients | KRT | CKD-EPI |
| Sheer(93) | Sheer | 2022 | American administrative claims data cohort | eGFR 15-59 or UACR ≥30mg/g | Primary & secondary care | KRT or CKD stage 5/eGFR <15ml/min/1.73m^2^ (>1 eGFR value on different dates) | Not reported |
| Smith(94) | Smith | 2013 | Academic Study cohort | eGFR15-59 | Secondary care study data | KRT | CKD-EPI |
| Stefan(95) | KFRE-4,  KFRE + renal chronicity score | 2020 | Canadian Biopsy cohort | Biopsy proven glomerular disease | Secondary care | KRT | CKD-EPI |
| Sud(96) | Sud | 2014 | Patients referred to secondary care nephrology clinic | eGFR<60 | Secondary care | KRT or all-cause mortality | CKD-EPI |
| Sun(97) | Sun | 2020 | Chinese biopsy cohort | Biopsy proven DKD + eGFR >30 | Secondary care | KRT or death due to chronic renal failure or ESRD | Not reported |
| Tangri(2) | KFRE-4, KFRE-6, KFRE-8 | 2016 | CKD prognosis consortium (CKD-PC) | eGFR<60 | Primary & secondary care | KRT | CKD-EPI 2009 |
| Tangri(98) | Dynamic model, KFRE=8 | 2017 | CKD patients referred to Canadian CKD outpatient clinic | eGFR <60 | Secondary care outpatient | KRT | CKD-EPI 2009 |
| Tangri(23) | KFRE-4, KFRE-6, KFRE-8 | 2011 | CKD patients referred to Canadian CKD outpatient clinic | eGFR <60 | Secondary care | KRT | CKD-EPI 2009 |
| Thanabalasingam(99) | Grams, KFRE-4 | 2022 | CKD patients referred to Canadian CKD MDT outpatient clinic | eGFR<60 | Secondary care | KRT | CKD-EPI |
| van den Brand(100) | KFRE-4  KFRE-4 + slope eGFR | 2019 | MASTERPLAN and NephroTest cohorts | eGFR 30-60 | Secondary care study data | KRT, death due to kidney failure or eGFR <15ml/min/1.73m^2^ | CKD-EPI 2009 |
| Wang(101) | KFRE-4 | 2019 | Singapore primary care cohort | eGFR <60 | Primary care | Serum creatinine ≥880 μmol/l, eGFR/CrCl <15ml/min/1.73m^2^, KRT | CKD-EPI, MDRD |
| Wang(102) | Wang | 2017 | Chinese biopsy cohort | Biopsy proven, glomerular disease, urine protein >0.3g/24hrs, eGFR≥15 | Secondary care | eGFR<15ml/min or KRT | CKD-EPI |
| Wang(103) | RRS | 2023 | Multicentre Chinese biopsy cohort | Biopsy proven ANCA GN | Secondary care | KRT | CKD-EPI, MDRD |
| Whitlock(104) | KFRE-4 | 2017 | Unreferred Canadian general primary care CKD cohort | eGFR<60 | Primary care | KRT | CKD-EPI |
| Wu(105) | Wu | 2022 | Chinese biopsy cohort | Biopsy proven MPO AAV | Secondary care | KRT or death | CKD-EPI |
| Xie(106) | Clinical Progression Risk Score,  Goto, RENAAL (Keane et al.), Berthoux models | 2012 | Chinese biopsy cohort | Biopsy proven IgAN | Secondary care | KRT | MDRD modified for Chinese population |
| Xie(107) | Xie | 2016 | American safety-net healthcare system, CKD cohort | eGFR<60, non-dialysis dependent | Primary & secondary care | KRT or death | MDRD |
| Xu(108) | Xu | 2021 | Dryad Digital Repository | CKD stage 2-5 + visited a nephrology centre | Secondary care study data | KRT or >50% decline in eGFR | Not reported |
| Yamanouchi(109) | KFRE-4, D-score, KFRE + D-score | 2018 | Multicentre Japanese biopsy cohort | Biopsy confirmed DKD, eGFR<60 | Secondary care | KRT or death from uraemia | MDRD |
| Yang(110) | MEST score + MMP7 | 2020 | Chinese biopsy cohort | Biopsy proven IgAN, eGFR≥30 | Secondary care | ≥40% decline in eGFR (two measurements at least 30 days apart), KRT, eGFR <15ml/min/1.73m^2^, KRT or death | CKD-EPI |
| Ye(111) | Ye | 2022 | CRIC study participants with T2DM | eGFR 20-70 | Secondary care study data | KRT | CKD-EPI |
| Yuan(112) | Yuan | 2020 | Chinese hospital CKD cohort | eGFR 30-60 (eGFR cut-off <43ml/min used in models) | Secondary care | Sustained eGFR<15ml/min/1.73m^2^ | CKD-EPI for Chinese patients |
| Zacharias(113) | KFRT risk model, KFRE-4 | 2022 | SKS, CKD-REIN, GCKD, MMKD cohorts | CKD stages 1-5 | Study data | KRT | CKD-EPI |
| Zacharias(114) | KFRE-4 plus NMR spectroscopy, KFRE-4 | 2019 | GCKD cohort | eGFR 30-60 or >60 + overt proteinuria | Secondary care | KRT | CKD-EPI |
| Zhang(115) | Zhang | 2022 | Chinese chronic disease management clinic cohort | eGFR<60 | Secondary care | KRT or death | CKD-EPI |
| Zhang(116) | IgA prediction tool (Barbour) | 2020 | Chinese biopsy cohort | Biopsy proven IgAN cohort |  | 50% decline in eGFR, eGFR <15ml/min/1.73m^2^ or KRT | CKD-EPI |
| Zhang(117) | Zhang LN model | 2021 | Chinese biopsy cohort | Biopsy proven LN | Secondary care | All-cause mortality, persistent decline in eGFR to 50% of baseline level, KRT | CKD-EPI |
| Zhang(118) | Zhang DKD model | 2021 | Chinese multicentre biopsy cohort | Biopsy proven DKD | Secondary care | KRT or all-cause mortality | CKD-EPI |
| Zhang(119) | KFRE-4 + pathology | 2023 | China Kidney Biopsy Cohort Study | Biopsy proven glomerular disease | Secondary care | KRT or ≥40% decrease in eGFR | CKD-EPI 2009 |
| Zhu(120) | Zhu dynamic model | 2020 | African American Study of Kidney Disease and Hypertension (AASK) study cohort | eGFR 20-65 and hypertension | Clinical trial | KRT or death | measured GFR |
| Zhu(121) | Zhu FSGS model | 2022 | Chinese biopsy cohort | Biopsy proven FSGS, eGFR≥15 | Secondary care | KRT or eGFR<15ml/min/1.73m^2^ | MDRD modified eGFR for Chinese patients |
| Zou(122) | Zou MLM | 2022 | Chinese biopsy cohort | Biopsy proven DKD, T2DM, eGFR>15 | Secondary care | KRT or eGFR<15ml/min/1.73m^2^ | CKD-EPI |

*Baseline comorbidity data reported for overall cohort from which study sample was taken, not reported for participants included in final model development/validation cohorts

AAV – ANCA associated vasculitis

MLM – machine learning model

DKD – diabetic kidney disease

T2DM – type two diabetes mellitus

Cox PH model – cox proportional-hazards model

MDT – multi-disciplinary team

**Additional file 1: Table S7. Summary of original models**

| Author | Year | Model | Number of variables | Variables |
| --- | --- | --- | --- | --- |
| Al-Wahsh | 2021 | Al-Wahsh | 15 | Age (10 years), age (squared term), male sex, log ACR, eGFR, CVD, diabetes, male sex*eGFR, CVD*eGFR, diabetes*eGFR, diabetes*log ACR, CVD*age, CVD*age squared, CVD*log ACR, CVD*diabetes. |
| Bai | 2021 | 5x MLM | Unclear | - |
| Barbour | 2016 | MEST score + clinical data | 3 (clinical data at biopsy model) | eGFR, proteinuria, MAP (at time of biopsy) |
|  |  |  | 3 (clinical data over 2 years model) | eGFR, proteinuria, average MAP (over 2 years) |
|  |  |  | 8 (clinical data + MEST model) | eGFR, proteinuria, MAP, MEST1T2 |
| Barbour | 2019 | IgA prediction tool | 3 (clinical model) | eGFR, MAP, proteinuria |
|  |  |  | 7 (clinical model + MEST) | eGFR, MAP, proteinuria, M1, E1, S1, T1/T2 |
|  |  |  | 13 (full model with race) | eGFR, MAP, proteinuria, M1, E1, S1, T1/T2, proteinuria x T1/T2, proteinuria x MAP, age, race, RASB, immunosuppression |
|  |  |  | 13 (full model without race) | eGFR, MAP, proteinuria, M1, E1, S1, T1/T2, proteinuria x T1/T2, proteinuria x MAP, age, RASB, immunosuppression, proteinuria x RASB |
| Bellocchio | 2021 | PROGRESS-CKD | 34 | age, gender, body mass index, smoking status, albumin, uACR, calcium, eGFR, slope of linear regression of eGFR over last 12 months, Haemoglobin, phosphate, urine protein, PTH, sodium, ferritin, aetiology of kidney disease (diabetes, hypertension, GN, PKD), comorbidities (cerebrovascular disease, chronic pulmonary disease, CHF, CTD, CAD, dementia, diabetes with organ damage, diabetes without complications, hemiplegia, hypertension, mild liver disease, mod-severe liver disease, PVD), number of hospitalisations, SBP |
| Belur | 2020 | 4x MLMs | 4 (final feed forward neural network model) | uACR, albumin, uric acid, creatinine |
|  |  |  | 5 (FNN 3) | uACR, albumin, phosphorus, Haemoglobin, creatinine |
|  |  |  | 7 (FNN 2) | age, uACR, serum albumin, serum uric acid, Haemoglobin, SBP and creatinine |
|  |  |  | 8 (FNN 1) | age, uACR, serum albumin, phosphorus, serum uric acid, Haemoglobin, SBP and creatinine |
| Chen | 2019 | Chen MLMs + cox regression | 3 (simplified scoring scale model) | tubular atrophy/interstitial fibrosis, global sclerosis, and urine protein excretion |
|  |  |  | 10 (XGBoost prediction model) | tubular atrophy/interstitial fibrosis (%), albumin, global sclerosis (%), hypertension before biopsy, uric acid, microscopic haematuria/RBC count, age, urine protein, mean mesangial score, creatinine |
| Cheng | 2020 | Cheng | 3 (Clinical model) | age, gender, oral hypoglycaemic drug use |
|  |  |  | 5 (Lab model) | haemoglobin, NLR, serum cystatin C, eGFR, 24-hour urine protein |
|  |  |  | 6 (Lab medication model) | haemoglobin, NLR, serum cystatin C, eGFR, 24-hour urine protein, hypoglycaemic drug use |
|  |  |  | 8 (Full model) | age, gender, haemoglobin, NLR, serum cystatin C, eGFR, 24-hour urine protein, and the use of oral hypoglycaemic drugs |
| Dai | 2021 | Dai | 12 | age, gender, CKD stage, CKD stage switch, hypertension, diabetes mellitus, hyperkalaemia, CHF, PVD, iron deficiency anaemia, prospective episode risk group score, poor adherence to RASB |
| Desai | 2011 | TREAT ESRD model + cardiac biomarkers | 17 (TREAT ESRD model) | age, sex, race, body mass index, insulin use, eGFR, urea, log uPCR, albumin, prior stroke, prior PVD, prior CHF, cardiac arrythmia, Haemoglobin, log ferritin, CRP, history of AKI |
|  |  |  | 18 (TREAT ESRD + cardiac biomarkers model) | age, sex, race, body mass index, insulin use, eGFR, urea, log uPCR, albumin, prior stroke, prior PVD, prior CHF, cardiac arrythmia, Haemoglobin, log ferritin, CRP, history of AKI, TnT/BNP levels (grouped as one) |
| Dimitrov | 2003 | Renal Risk Index | 3 | serum creatinine concentration (>2.4 mg/dL), 24hr UPE rate (>=3g/dl), Calcium*Phosphate product (>=32.64 mg/dl) |
| Edmonston | 2019 | Edmonston +FGF23 | 11 (Edmonston model) | age, sex, race, diabetes, CHF, hypertension medications, SBP, cholesterol, eGFR, albumin, uACR |
|  |  |  | 12 (Edmonston model + FGF23) | age, sex, race, diabetes, CHF, hypertension medications, SBP, cholesterol, eGFR, albumin, uACR, FGF23 |
| Forsblom | 2014 | Forsblom | 3 (model 1) | eGFR (2 fractional polynomial), duration of type 1 diabetes mellitus (linear form), HbA1C |
|  |  |  | 4 (model 2) | eGFR (2 fractional polynomial), duration of type 1 diabetes mellitus (linear form), HbA1C, TNFalphaR1 (-0.5 fractional polynomial)) |
| Gibertoni | 2019 | CT-PIRP | 6 | gender, age, proteinuria, baseline eGFR, phosphate levels, diabetes |
| Grams | 2018 | Markov model | 30 | Age, sex, race, eGFR, albumin-to-creatinine ratio, systolic blood pressure, smoking status, diabetes mellitus, history of CVD, age*male, age*black race, age CVD, age*smoker, age*SBP, age* diabetes, age*eGFR, age*uACR, CVD*male, CVD*black race, CVD*SBP, CVD*eGFR, CVD*uACR, smoker*black race, smoker*SBP, smoker*diabetes, smoker*eGFR, smoker*uACR, diabetes*eGFR, diabetes*uACR, eGFR*uACR |
| Hasengawa | 2019 | Hasegawa | 8 | age, sex, eGFR, albuminuria, SBP, diabetes, serum albumin + Haemoglobin |
| Hoshino | 2015 | Hoshino | 3 (model 1) | age, eGFR, proteinuria |
|  |  |  | 7 (model 2) | Proteinuria, eGFR, pathological score of diabetic nephropathy (D- score) [glomerular classes, tubular atrophy/interstitial fibrosis, interstitial inflammation, hyalinosis, arteriosclerosis] |
|  |  |  | 8 (model 3) | age, eGFR, urinary protein + (D-score) |
| Hsu | 2017 | Hsu | 12 (Base model) | age, sex, race/ethnicity, clinical centre, albumin/ creatinine ratio, estimated glomerular filtration rate; diabetes mellitus; cardiovascular disease; systolic blood pressure, body mass index, angiotensin-converting enzyme inhibitor/angiotensin receptor blocker use, and education |
|  |  |  | 13 (Base model plus urine biomarker) | age, sex, race/ethnicity, clinical centre, albumin/ creatinine ratio, estimated glomerular filtration rate; diabetes mellitus; cardiovascular disease; systolic blood pressure, body mass index, angiotensin-converting enzyme inhibitor/angiotensin receptor blocker use, education and urine biomarker |
| Jiang | 2019 | Jiang | 4 | urinary protein excretion, CKD stage, extra capillary hypercellularity, glomerular hyalinosis |
| Johnson | 2008 | Johnson 2008 | 6 | age, sex, eGFR, diabetes, anaemia, hypertension |
| Johnson | 2007 | Johnson 2007 | 6 | age, sex, hypertension, diabetes, eGFR, anaemia |
| Kwan | 2020 | Kwan | 9 (clinical model) | Baseline data of age, race, sex, smoked >100 cigarettes in lifetime, body mass index, HbA1c, mean arterial pressure, urine albumin, and eGFR |
|  |  |  | 13 (metabolite model) | aconitic acid, citric acid (nucleotide metabolism: tricarboxylic acid [TCA] cycle); uracil (nucleotide metabolism: purine); 3-hydroxyisobutyrate, 2-methylacetoacetate, 3- hydroxyisovalerate, 2-ethyl-3-hydroxypropionate, 3-methylcrotonyglycine, tiglyglycine (amino acid metabolism: valine, leucine, and isoleucine); homovanillic acid (amino acid metabolism: phenylalanine and tyrosine); glycolic acid, 3-methyladipic acid, and 3- hydroxypropionate |
|  |  |  | 22 (clinical metabolite model) | Baseline data of age, race, sex, smoked >100 cigarettes in lifetime,body mass indexI, HbA1c, mean arterial pressure, urine albumin, and eGFR + aconitic acid, citric acid (nucleotide metabolism: tricarboxylic acid [TCA] cycle); uracil (nucleotide metabolism: purine); 3-hydroxyisobutyrate, 2-methylacetoacetate, 3- hydroxyisovalerate, 2-ethyl-3-hydroxypropionate, 3-methylcrotonyglycine, tiglyglycine (amino acid metabolism: valine, leucine, and isoleucine); homovanillic acid (amino acid metabolism: phenylalanine and tyrosine); glycolic acid, 3-methyladipic acid, and 3- hydroxypropionate |
| Landray | 2010 | Landray | 4 | log creatinine, log phosphate, log uACR, and sex |
| Lee | 2018 | Lee | 4 (CKD stage 5 model) | haemoglobin, urea, MDRD eGFR, urinary protein |
|  |  |  | 10 (CKD stage 4 model) | age, sex, diabetes mellitus, GN, PKD, haemoglobin, urea, calcium, MDRD eGFR, urinary protein |
|  |  |  | 10 (CKD stage3 model) | age, sex, DM, PKD, albumin, haemoglobin, phosphorus, potassium, CKD-EPI eGFR, urinary protein |
| Lim | 2019 | Lim | 3 (model 1) | age, sex, eGFR |
|  |  |  | 4 (model 2) | age, sex, eGFR, uACR |
|  |  |  | 5 (model 3) | age, sex, eGFR, uACR, race |
|  |  |  | 6 (model 4) | age, sex, eGFR, uACR, diabetes, hypertension |
|  |  |  | 6 (model 5) | age, sex, eGFR, uACR, diabetes, hyperlipidaemia |
| Lin | 2023 | Lin | 12 | female sex, race, smoking status, age at type 2 diabetes mellitus, diagnosis, SBP, heart rate, HbA1c, eGFR, uACR, retinopathy event occurring in last year, antihypertensive drug use, interaction term between SBP and female |
| Maziarz | 2015 | Maziarz | 4 (model 1) | age, sex, race-ethnicity and eGFR |
|  |  |  | 6 (model 2) | age, sex, race-ethnicity, eGFR, dipstick proteinuria and an interaction between eGFR and dipstick |
|  |  |  | 12 (model 3) | age, sex, race-ethnicity, eGFR, dipstick proteinuria and an interaction between eGFR and dipstick, health insurance coverage, comorbidities (diabetes mellitus, CVD, hypertension, substance abuse and chronic viral disease) |
|  |  |  | 16 (model 4) | age, sex, race-ethnicity, eGFR, dipstick proteinuria and an interaction between eGFR and dipstick, health insurance coverage, comorbidities (diabetes mellitus, CVD, hypertension, substance abuse and chronic viral disease), additional laboratory variables (albumin, calcium, haemoglobin and cholesterol) |
| Maziarz | 2014 | Maziarz2 | 4 (model 1) | age, sex, race-ethnicity and eGFR |
|  |  |  | 6 (model 2) | age, sex, race-ethnicity, eGFR, dipstick proteinuria and an interaction between eGFR and dipstick |
|  |  |  | 12 (model 3) | age, sex, race-ethnicity, eGFR, dipstick proteinuria and an interaction between eGFR and dipstick, health insurance coverage, comorbidities (diabetes mellitus, CVD, hypertension, substance abuse and chronic viral disease) |
|  |  |  | 16 (model 4) | age, sex, race-ethnicity, eGFR, dipstick proteinuria and an interaction between eGFR and dipstick, health insurance coverage, comorbidities (diabetes mellitus, CVD, hypertension, substance abuse and chronic viral disease), additional laboratory variables (albumin, calcium, haemoglobin and cholesterol) |
| Orlandi | 2018 | Orlandi | 11 (model 1) | age, race, sex, eGFR, albuminuria, diabetes, systolic blood pressure, body mass index, waist circumference, NT-pro-BNP, serum albumin |
|  |  |  | 12 (model 2) | age, race, sex, eGFR, albuminuria, diabetes, systolic blood pressure, body mass index, waist circumference, NT-pro-BNP, serum albumin, haematuria |
|  |  |  | 13 (model 3) | age, sex, eGFR, albuminuria, diabetes, waist circumference, NT-pro-BNP, FGF-23, Calcium, PTH, serum albumin, uric acid, triglycerides |
|  |  |  | 14 (model 4) | age, sex, eGFR, albuminuria, diabetes, waist circumference, NT-pro-BNP, FGF-23, Calcium, PTH, serum albumin, uric acid, triglycerides, haematuria |
| Pesce | 2016 | Pesce | 6 | Gender, age, histological grading, serum creatinine, 24hr proteinuria, hypertension |
| Schena | 2021 | Schena (CDSS - ANN model) | 13 | age, sex, SBP, DBP, serum creatinine MESTC, 24-hour proteinuria, RASB, immunosuppression |
| Schroeder | 2017 | Schroeder KPNW score | 8 | age, sex, eGFR, haemoglobin, presence of proteinuria or albuminuria, systolic blood pressure, antihypertensive use, and the Diabetes Complications Severity Index |
| Sheer | 2022 | Sheer | 21 (logistic regression with backward elimination model) | age, sex, race, CHF, PVD, hypertension (complicated), COPD, fluid/electrolyte disorder, complicated diabetes, liver failure, anaemia, smoking, eGFR category, uACR category, LDL category, HDL category, serum creatinine, HbA1c, adherence to insulin, diuretics, physician encounters |
|  |  |  | 22 (LASSO model) | sex, race, CHF, PVD, hypertension (complicated), COPD, fluid/electrolyte disorder, complicated diabetes, liver failure, anaemia, smoking, eGFR category, uACR category, serum creatinine, HbA1c, metformin, insulin, statins, beta blockers, calcium channel blockers, diuretics, physician encounters |
| Smith | 2013 | Smith | 5 | CVD, eGFR, triglyceride, uPCR, uNCR |
| Sud | 2014 | Sud | 12 | age, sex, DBP, eGFR, albumin, phosphate, bicarbonate, urea nitrogen, albuminuria, HF, hypertension, diabetes |
| Sun | 2020 | Sun | 4 (clinical model) | cystatin C, eGFR, BNP, log uACR |
|  |  |  | 5 (clinical-pathological model) | Pathological grade, cystatin C, eGFR, BNP, log uACR |
|  |  |  | 5 (clinical-medication model) | RASB use, cystatin C, eGFR, BNP, log uACR |
|  |  |  | 7 (full model) | Age, pathological grade, RASB use, cystatin C, eGFR, BNP, log uACR |
| Tangri | 2011 | KFRE | 3-var KFRE | age, sex, eGFR |
|  |  |  | 4-var KFRE | age, sex, log uACR, eGFR |
|  |  |  | 6-var KFRE | age, sex, log uACR, eGFR, diabetes, hypertension |
|  |  |  | 8-var KFRE | age, sex, eGFR, uACR, albumin, phosphate, bicarbonate, calcium |
| Tangri | 2017 | Dynamic model | 8 | age, sex, eGFR, uACR, albumin, phosphate, bicarbonate, calcium (dynamic laboratory measures except uACR) |
| van den Brand | 2019 | van den Brand – cox PH model | 4 (cox PH model with time varying eGFR) | age, sex, uACR, eGFR at 2 year follow up |
|  |  |  | 5 (cox PH model with slope eGFR) | age, sex, uACR, eGFR at landmark time and eGFR slope |
|  |  |  | 6 (shared parameter joint model) | sex, age, uACR, current eGFR, eGFR slope + follow up time |
| Wang | 2017 | Wang | 5 (model 1) | eGFR, urine protein, blood pressure, age, sex |
|  |  |  | 6 (model 2) | urinary fibrinogen, eGFR, urine protein, blood pressure, age, sex |
| Wu | 2022 | Wu | 3 (clinical model) | age, eGFR, proteinuria |
|  |  |  | 4 (clinical model +MMP7) | age, eGFR, proteinuria, uMMP7 |
|  |  |  | 6 (clinpath model) | age, eGFR, proteinuria, % normal glomeruli, tubular atrophy/interstitial fibrosis |
|  |  |  | 7 (clinpath model + MMP7) | age, eGFR, proteinuria, % normal glomeruli, tubular atrophy/interstitial fibrosis, uMMP7 |
| Xie | 2012 | Clinical Progression Risk Score | 4 | eGFR, Haemoglobin, serum albumin, SBP |
| Xie | 2016 | Xie | 5 (model 1) | eGFR, dipstick proteinuria, residual associations with age, sex, and race-ethnicity |
|  |  |  | 11 (model 2) | eGFR, dipstick proteinuria, residual associations with age, sex, and race-ethnicity, health insurance coverage, comorbidities (diabetes mellitus, CVD, hypertension, substance abuse and chronic viral disease) |
|  |  |  | 15 (model 3) | eGFR, dipstick proteinuria, serum albumin, calcium, cholesterol and haemoglobin, residual associations with age, sex, and race-ethnicity, health insurance coverage, comorbidities (diabetes mellitus, CVD, hypertension, substance abuse and chronic viral disease) |
| Xu | 2021 | Xu | 5 | Disease aetiology, proteinuria (+/-), Haemoglobin, creatinine, uPCR |
| Yamanouchi | 2018 | D-score | 5 | glomerular lesions, tubular atrophy/interstitial fibrosis, interstitial inflammation, arteriolar hyalinosis, arteriosclerosis |
| Yang | 2020 | MEST score + MMP7 | 3 (clinical model) | MAP, proteinuria, eGFR |
|  |  |  | 8 (clinical model + MEST-C) | MAP, proteinuria, eGFR, MEST-C |
|  |  |  | 9 (clinical model + MEST-C + urinary biomarker) | MAP, proteinuria, eGFR, MEST-C, urinary biomarker (5 different biomarkers added individually) |
|  |  |  | 13 (clinical model + MEST-C + all urinary biomarkers) | MAP, proteinuria, eGFR, MEST-C + uMMP7 + AGF, + EGF + KIM1 + serum Gd-IgA1 |
| Ye | 2022 | Ye | 8 | age at diabetes diagnosis, sex, body mass index, SBP, eGFR, uPCR, CHF, myocardial infarction |
| Yuan | 2020 | Yuan | 30 (CKD3A model) | Albumin, proteinuria, total protein, total bilirubin, direct bilirubin, albumin-to-globulin ratio, haemoglobin, serum calcium, eGFR, blood haematocrit, total cholesterol, ALT, HDL, urine specific gravity, serum creatinine, urea, age, blood glucose, red blood cells, potassium, LDL, triglycerides, lymphocyte percentage, diabetes, neutrophil percentage, monocyte percentage, eosinophil percentage, mean corpuscular haemoglobin concentration, urine glucose, urine pH |
|  |  |  | 30 (CKD3B model) | Serum creatinine, eGFR, total protein, total cholesterol, urea, eosinophil percentage, albumin, mean corpuscular haemoglobin, total bilirubin, diabetes, AST, eosinophil count, sodium, HDL, chloride, proteinuria, age, direct bilirubin, LDL, urine glucose, MCV, triglycerides, lymphocyte count, globulin, HDL:total cholesterol ratio, red blood cell volume distribution, glucose, platelet volume distribution width, red blood cell count , haemoglobin |
| Zacharias | 2022 | KFRT risk model | 6 | serum creatinine, serum cystatin C, UACR, serum urea, Haemoglobin, serum albumin (all log transformed) |
| Zhang | 2022 | Zhang | 3 (model A) | Age, eGFR, urine protein |
|  |  |  | 8 (model B) | Age, eGFR, urine protein, haemoglobin, serum uric acid, cardiovascular disease, primary disease, chronic disease management adherence |
|  |  |  | 8 (model C) | Age, eGFR, urine protein, alpha-blockers, beta-blockers, calcium supplements, Chinese herbal decoction, Chinese patent medicines for dispelling turbidity |
|  |  |  | 13 (model D) | Age, eGFR, urine protein, haemoglobin, serum uric acid, cardiovascular disease, primary disease, chronic disease management adherence, alpha-blockers, beta-blockers, calcium supplements, Chinese herbal decoction, Chinese patent medicines for dispelling turbidity |
| Zhang | 2021 | Zhang LN model | 8 | Haemoglobin, eGFR, serum albumin, sex, ISN/RPS class, tubular atrophy/interstitial fibrosis, proteinuria (tertiles), proteinuria/albumin interaction |
| Zhang | 2021 | Zhang DKD model | 3 (model 1) | Baseline eGFR, urine proteinuria, albumin |
|  |  |  | 3 (model 2) | Modified arteriosclerosis score. Baseline eGFR and urine proteinuria. |
| Zhu | 2020 | Zhu dynamic model | 8 | Four longitudinal biomarkers (eGFR, urine protein, albumin, SBP) plus gender, age at time of prediction, eGFR slope and blood pressure volatility |
| Zhu | 2022 | Zhu FSGS model | 3 | eGFR, haematuria, percentage of sclerosis |
| Zou | 2022 | Zou MLM | 5 | cystatin C, Haemoglobin, serum albumin, urinary total protein, eGFR |
| Berthoux*(123) | 2011 | ARR | 3 | hypertension, proteinuria of≥1 g/24 h and severe pathologic lesions |
| Brix*(124) | 2018 | Renal Risk Score | 3 | Normal glomeruli (%), tubular atrophy/interstitial fibrosis (%), renal function at time of diagnosis (eGFR) |
| Cattran*(125) | 2009 | Oxford classification | 4 | Mesangial hypercellularity score, segmental glomerulosclerosis, endocapillary proliferation, tubular atrophy/interstitial fibrosis |
| Goto*(126) | 2009 | Goto | 8 | Sex, age, SBP, proteinuria, haematuria, serum albumin, eGFR, histological grade |
| Haas*(127) | 1997 | Haas classification | 5 | Mesangial hypercellularity, glomerular sclerosis, focal proliferative GN, diffuse proliferative GN, tubular atrophy/loss |
| Kawamura*(128) | 2013 | Japanese histologic classification 2013 | 4 (model 1) | glomerular lesion percentage score, initial MAP, proteinuria, eGFR, |
|  |  |  | 6 (model 2) | glomerular lesion percentage score, initial MAP, proteinuria, eGFR, immunosuppressive therapy, RASB |
| Keane*(129) | 2006 | RENAAL | 4 | Mesangial hypercellularity, segmental glomerulosclerosis, endocapillary hypercellularity, tubular atrophy/interstitial fibrosis |
| Marks*(130) | 2015 | Marks | 4 | Age, sex, eGFR, proteinuria |
| Sethi*(131) | 2017 | Renal chronicity score | 4 | Glomerulosclerosis, interstitial fibrosis, tubular atrophy, arteriolosclerosis |
| Tanaka*(132) | 2013 | Tanaka | 5 | Urinary protein excretion, eGFR, MST |

*Original study developing model not included in the review

MEST - mesangial hypercellularity (M), endocapillary hypercellularity (E), segmental glomerulosclerosis (S), tubular atrophy/interstitial fibrosis (T)

AAV – ANCA associated vasculitis

MLM – machine learning model

DKD – diabetic kidney disease

T2DM – type two diabetes mellitus

Cox PH model – cox proportional-hazards model

MDT – multi-disciplinary team

MAP – mean arterial pressure

RASB – renin-angiotensin system blockade

PTH – parathyroid hormone

CHF – congestive heart failure

CTD – connective tissues disease

CAD – coronary artery disease

SBP – systolic blood pressure

RBC – red blood cell

NLR – neutrophil to lymphocyte ratio

PVD – peripheral vascular disease

CRP – c-reactive protein

AKI – acute kidney injury

TnT – troponin T

BNP – brain natriuretic peptide

UPE – urine protein excretion

FGF23 – fibroblast growth factor 23

HbA1C – glycated haemoglobin

TNFalphaR1 – tumour necrosis factor-α receptor 1

GN – glomerulonephritis

PKD – polycystic kidney disease

NT–pro-BNP – N-terminal pro-B-type natriuretic peptide

DBP – diastolic blood pressure

COPD – chronic obstructive pulmonary disease

uPCR – urine protein-to-creatinine ratio

uNCR – urinary neutrophil gelatinase-associated lipocalin-to-creatinine ratio

uMMP7 – urinary matrix metalloproteinase 7

AGT – angiotensinogen

EGF – epidermal growth factor

K1M1 – kidney injury molecule 1

Gd-IgA1 – galactose-deficient immunoglobulin A1

ALT – alanine aminotransferase test

HDL – high density lipoprotein

LDL – low density lipoprotein

MCV – mean cell volume

ISN/RPS – International Society of Nephrology and the Renal Pathology Society

MST – mesangial hypercellularity (M), segmental glomerulosclerosis (S), tubular atrophy/interstitial fibrosis (T)

**Additional file 1: Table S8. Baseline characteristics of included studies**

| Author | Year | Participants (n) | Outcome events (n) | Death events (n) | Female n (%) | Age  Mean (SD) | eGFR (mean) | Diabetes (%) | Hypertension (%) | CVD (%) |
| --- | --- | --- | --- | --- | --- | --- | --- | --- | --- | --- |
| Ali | 2021 | 743 | 331 | 164 | 281 (37.8) | 67.5 (15.0) | 15.7 (3.7) | 39.8 | 97.3 | - |
| Ali 2021 2-year validation |  | 743 | 257 | 101 | - |  |  | - | - | - |
| Al-Wahsh | 2021 | 14619 | 3265 | 6582 | 7549 (51.6) | 74.1 (12.8) | 27.2 (2.9) | 67.6 | - | 56.7 |
| Al-Wahsh 2021 validation |  | 2295 | 722 | 875 | 1143 (49.8) | 71.9 (14) | 24.7 (3.7) | - | - | 34.7 |
| Bai | 2022 | 748 | 70 | Not reported | 329 (44.0) | 57.8 (17.6) | 48.8 (26.1) | 55.5 | 74.6 | 23.7 |
| Bai | 2021 | 65 | 34 | 8 | 33 (50.8) | 61.8 (10.2) | 12.9 (11.5) | - | - | - |
| Barbour | 2022 | 2507 | 385 | Not reported | 1033 (41.2) | 37 (12.6) | 82.7 (37.8) | - | - | - |
| Barbour 2022 validation |  | 722 | 123 | Not reported | 324 (44.9) | 37 (12.6) | 81 (31.9) | - | - | - |
| Barbour | 2019 | 2781 | 492 | 35 | 1173 (42.2) | 36.4 (12.8) | 82.7 (38.0) | - | - | - |
| Barbour 2019 validation |  | 1146 | 213 | 0 | 581 (50.7) | 35.6 (13.4) | 89.2 (35.2) | - | - | - |
| Barbour | 2016 | 901 | 162 | Not reported | 261 (29.0) | 38.8 (14.9) | 68.5 (29.8) | - | - | - |
| Bellocchio | 2021 | 9407 | 1817 | Excluded | - | - | - | - | - | - |
| Bellocchio 2021 validation |  | 3684 | 80 | Excluded | - | - | - | - | - | - |
| Belur | 2020 | 11789 | 1280 | Not reported | - | - | - | - | - | - |
| Bon | 2023 | 473 | 98 | 32 | 127 (26.8) | 37.3 (17.1) | 84.7 (30.8) | - | - | - |
| Bundy | 2022 | 3873 | 856 | Not reported | 1751 (45.2) | 57.8 (10.9) | 44.4 (25.3) | 48.2 | 91.3 |  |
| Chen | 2019 | 1022 | 74 | Not reported | 496 (48.5) | 34.6 (9.4) | 90.7 (29.2) | 0 | 42.8 | - |
| Chen 2019 validation |  | 1025 | 114 | Not reported | 512 (50.0) | 35 (9.8) | 85.2 (31.7) | 0 | 32.8 | - |
| Cheng | 2020 | 641 | 272 | Excluded | 260 (40.6) | 56.0 (11.9) | 56.6 (72.4) | 100 | 70.0 | 9.0* |
| Cheng 2020 validation |  | 280 | 138 | Excluded | 167 (59.6) | 51.7 (10.4) | 55.4 (33.9) | 100 | 79.6 | 11.8* |
| Chu | 2023 | 1641 | 268 | 180 | 799 (48.7) | 68.6 (13) | 28.3 (12.6) | 57.8 | 91.8 | 31.7* |
| da Silva | 2023 | 256 | 159 | 40 | 91 (35.5) | 70.4 (12.9) | 16.1 (10.4) | - | - | - |
| da Silva | 2022 | 360 | 23 | 86 | 164 (45.6) | 74.9 (12.2) | 33.4 (12.1) | 45.3 | 90.6 | 47.8 |
| Dai | 2021 | 74114 | 2476 | Excluded | 42419 (57.2) | 74.4 (9.7) | - | 40.0 | 89.7 | 27.1* |
| Desai | 2011 | 1000 | 222 | 185 | 558 (55.8) | 67.7 (10.7) | 34.9 (11.7) | 100 | - | 38.8* |
| Dimitrov | 2003 | 344 | 80 | Not reported | - | - | - | - | - | - |
| Edmonston | 2019 | 3879 | 929 | 823 | 1737 (44.8) | 57.7 | 42.8 (13.5) | 48.5 | 86.1 | 22.0 |
| Fenton | 2018 | 556 | 60 | 34 - excluded from analysis | 205 (36.9) | 63.7 (18.6) | 26 (11.1) | 35.3 | - | 21.6* |
| Floyd | 2023 | 174 | 106 | 53 - unclear how many before or after ESKD | 99 (56.9) | 58 (18.7) | 8.2 (6.0) | - | - | - |
| Forsblom | 2014 | 459 | 130 | 59 | 200 (43.6) | 41.6 (10.6) | 57.7 (28.4) | 100 | 92.6 | 19.0* |
| Gibertoni | 2019 | 2265 | 536 | 657 | 790 (34.9) | 71.2 (12.9) | 29 (13.1) | 32.6 | - | - |
| Gibertoni 2019 validation |  | 2051 | not reported | not reported | 679 (33.1) | 74.8 (13) | 29.6 (18.7) | 38.1 | - | - |
| Grams | 2018 | 264296 | 31541 | 123985 |  |  |  | - | - | - |
| Grams | 2023 | 91578 | 4609 | Not reported | 49452 (54.0) | 73 (11) | 43 (13) | - |  | 36.0* |
| Grams 2023 validation |  | 142591 | 3693 | Not reported | 65592 (46.0) | 74 (11) | 45 (12) | - | - | 32.0* |
| Haaskjold | 2023 | 306 | 151 | 17 | 72 (23.5) | 37.4 (14) | 79.3 (32.6) | - | - | - |
| Hallan | 2019 | 1188 | 42 | 462 | 675 (56.8) | 79.9 (6.8) | 35.8 (7.8) | 17.2 | - | 22.8* |
| Hasengawa | 2019 | 1017 | 206 | 27 | 375 (36.9) | 60.6 (11.6) | 28.8 (12.5) | 38.7 | 84.0 | - |
| Hasengawa 2019 validation |  | 1017 | 216 | 30 | 359 (35.3) | 61.1 (11.1) | 27.5 (12.1) | 91.5 | 84.0 | - |
| Hoshino | 2015 | 205 | unclear 121 | Not reported | 55 (26.8) | 55.9 (13) | 44.4 (22.9) | 100 | - | - |
| Hsu | 2017 | 2466 | 581 | Not reported | 1131 (45.9) | 59.5 (10.8) | 43.6 (17.8) | 49.7 | - | 34.4 |
| Hundemer | 2020 | 1293 | 541 | 144 | 501 (38.7) | 68 (14.8) | 15.3 (5.2) | - | - | - |
| Hundemer 2-year KFRE cohort |  | 637 | 406 | 111 | 245 (38.5) | 67.3 (14.9) | 15 (5.9) | - | - | - |
| Hwang | 2021 | 545 | 53 | Not reported | 250 (45.9) | 40.0 (14.1) | 88.1 (31.7) | 5.0 | 32.1 | - |
| Ingwiller | 2022 | 314 | 157 | Excluded | 109 (34.7) | 67.1 (13.0) | - | 50 | - | - |
| Irish | 2023 | 12861 | 285 | 2607 | 6635 (51.6) | 70.7 (10.4) | 50.3 (9.3) | 55.0 | 41.2 | - |
| Irish | 2023 | 10429 | 276 | Not reported | 5314 (51.0) | 70.8 (10.6) | 49.9 (9.6) | 58.9 | 50.8 | - |
| Irish | 2023 | 4969 | 202 | Not reported | 2479 (49.9) | 68.3 (11.9) | 48.3 (11) | 53.0 | 48.6 | - |
| Jahan | 2023 | 406 | 71 | 112 | 195 (48.0) | 70.9 (12.2) | 30.9 (11.7) | 53.9 | - | - |
| Jiang | 2019 | 110 | 48 | 2 | 31 (28.1) | 52.0 (9.5) | - | 100 | 91.8 | 25.5 |
| Johnson | 2008 | 9782 | 323 | 3130 | 6184 (63.2) | 73 (10) | - | 27.6 | 90.2 | - |
| Johnson | 2007 | 6541 | 369 | 2678 | 4091 (62.5) | 74 (-) | - | 31.3 | 85.2 | - |
| Kang | 2020 | 13244 | 3706 | Not reported | 5562 (42.0) | 59.9 (14.6) | 36.1 (17.1) | 29.0 | 47.4 | - |
| Knoop | 2015 | 1134 | 251 | 69 | 305 (26.9) | 38 (16) | - | - | 36.2 | - |
| Kong | 2023 | 255 | 73 | Not reported | 119 (46.7) | 58.9 (8.4) | - | 12.5 | 48.2 | - |
| Kwan | 2020 | 1001 | 359 | 159 | 437 (43.7) | 59.9 (9.4) | 40.6 (11.2) | 100 | 92.5 | - |
| Kwek | 2022 | 1128 | 252 | 383 | 475 (42.1) | 67.1 (11.8) | 33.0 (16.6) | 64.6 | - | - |
| Landray | 2010 | 382 | 190 | 150 | 134 (35.1) | 61.5 (14.3) | 21.8 (10.7) | 17.3 | - | 44.8 |
| Landray 2010 validation |  | 213 | 66 | 65 | 76 (35.7) | 65.1 (13.5) | 21.6 (13.6) | 26.8 | - | 43.2 |
| Lee | 2018 | 1625 | 530 | Not reported | 897 (55.2) | 57.3 (10.6) | 39.4 (15.7) | 39.4 | 37.5 | - |
| Lee 2018 validation |  | 1618 | 473 | Not reported | 879 (54.3) | 58.5 (11.6) | 40.6 (25.6) | 38.7 | 34.7 | - |
| Lennartz | 2016 | 403 | 52 | Not reported | 168 (41.7) | 64.6 (12.6) | 45.8 (16) | 37.2 | - | - |
| Lennartz 2016 |  | 370 | 49 | Not reported | - | - | - | - | - | - |
| Lennartz 2016 validation |  | 162 | 23 | Not reported | 67 (41.4) | 49.8 (16.4) | 80.2 (47.6) | 13.0 | - | - |
| Lim | 2019 | 1970 | 32 | Not reported | 1050 (53.3) | 62.4 (10.2) | 76.0 (24.2) | 44.8 | 77.9 | - |
| Lin | 2023 | 6982 | 312 | Not reported | 2629 (37.7) | 66.1 (7.4) | 79.2 (28.5) | 100 | - | 24.9* |
| Lin validation cohort 2 |  | 2954 | 545 | Not reported | 1051 (35.6) | 67.4 (8.1) | 55.0 (15.3) | 100 | - | 99.9* |
| Lin validation cohort 3 |  | 1907 | 670 | Not reported | 844 (44.3) | 52 (9.8) | 40.7 (12.9) | 100 | - | 17.4* |
| Maher | 2023 | 27017 | 290 | 5421 | 15717 (58.2) | 76.6 (10.3) | 48 (9.9) | 30.54 | 70.3 | 33.2 |
| Maher cohort 2 |  | 2728 | 104 | 230 | 1442 (52.9) | 70.2 (11.6) | 48.1 (10.6) | 53.2 | 69.7 | 29.0 |
| Major | 2019 | 35539 | 429 | death rate 55.9 per 1000 person years (95% CI 54.8-57.) | 20436 (57.5) | 75.9 (10.6) | 48.2 (9.8) | 31.5 | 41.7 | 32.0 |
| Major 2 years |  | 35539 | 176 | death rate 55.9 per 1000 person years (95% CI 54.8-57.) | - | - | - | - | - | - |
| Massy | 2023 | 1000 | 262 | 108 | 310 (31.0) | 69 (11.9) | 28 (11) | 43 | 92 | 57 |
| Massy 2023 validation |  | 326 | 28 | 61 | 130 (40.0) | 70.3 (13.4) | 36 (13) | 38.0 | - | 45.1 |
| Maziarz | 2015 | 28779 | 1730 | Not reported | 14876 (51.7) | 60.3 (14.2) | - | 22.8 | 47.0 | 27.6 |
| Maziarz | 2014 | 982 | 71 | Not reported | 314 (32.0) | 49.6 (10.9) | - | 15.6 | 31.1 | 15.0 |
| Maziarz 2015 domiciled cohort |  | 15564 | 888 | Not reported | 8452 (54.3) | 59.3 (13.6) | - | 22.9 | 48.5 | 19.4 |
| Naranjo | 2021 | 976299 | 29653 | Death incidence per 1000 person years 69.28 (69.02-69.53) | 583827 (59.8) | 73 (10) | 46 (11) | 22.7- | 49.4 | - |
| Orlandi | 2018 | 3272 | 1071 | 480 | 1405 (42.9) | 57 (11) | 44 (16) | 50.5 | 88.4 | 22.8* |
| Ouyang | 2021 | 2300 | 288 | 10 | 1194 (51.9) | 35.7 (11.9) | 76.9 (39.7) | - | - | - |
| Park | 2014 | 500 | 52 | Not reported | 285 (57.0) | 37.1 (12) | 87.3 (28.5) | - | 24.4 | - |
| Peeters | 2013 | 595 | 114 | 59 | 184 (30.9) | 60.8 (12.1) | 33.3 (11.7) | 25.0 | - | 30.1 |
| Pesce | 2016 | 1040 | 241 | Not reported | 321 (30.9) | 34.9 (13.4) | 86.8 (27.4) | - | 41.8 | - |
| Prouvot | 2021 | 573 | 287 | 238 | 246 (42.9) | 82.3 (5.2) | 12.7 (3.7) | 39.4 | 97.9 | 31.4* |
| Ramspek | 2021 | 1580 | 458 | 330 | 545 (34.5) | 76.1 (8.0) | 18.5 (4.7) | 42.1 | 91.7 | 62.2 |
| Ramspek 2021 - SRR cohort |  | 13489 | 2764 | 3357 | 5220 (38.7) | 73.7 (11.5) | 21.9 (5.7) | 36.4 | 73.2 | 33.1 |
| Sato | 2015 | 198 | 51 | 0 | 103 (52.0) | 42 (10.1) | 71.3 (25.9) | - | 42.9 | - |
| Schena | 2021 | 948 | 210 | Not reported | 263 (27.7) | 40.6 (14) | 67.3 (33.4) | - | 30.3 | - |
| Schena 2021 - Thessaloniki cohort |  | 167 | 23 | Not reported | 47 (28.1) | 40.1 (15.5) | 69.6 (31.6) | - | 34.7 | - |
| Schroeder | 2017 | 22460 | 737 | Not reported | 13378 (59.7) | 74.6 (10.1) | 46.8 (10.1) | 34.3 | 85.1 | - |
| Schroeder 2017 - validation cohort |  | 16553 | 360 | Not reported | 10270 (62.0) | 74.7 (9) | 47.5 (9.8) | 27.0 | 81.9 | - |
| Sheer | 2022 | 169876 | 7928 | Not reported | 90841 (53.5) | 75.2 (6.1) | 47.9 (9.7) | 100 | 21.3 | - |
| Smith | 2013 | 158 | 40 | 20 | 40 (25.3) | 69 (12) | 32 (11) | 25.3 | - | 46.8 |
| Stefan | 2020 | 625 | 78 | Not reported | 269 (43.0) | 46.7 (14.6) | 55.9 (29.6) | - | 50.1 | - |
| Sud | 2014 | 3273 | 459 | 540 | 1426 (43.6) | 70 | 36 (13) | 50.0 | 75.7 | 41.9* |
| Sun | 2020 | 478 | 225 | Not reported | 186 (38.9) | 51.7 (10.4) | - | 100 | - | - |
| Tangri | 2016 | 721357 | 23829 | Not reported | 167974 (23.3) | 74 (10) | 46 (11) | - | - | - |
| Tangri | 2017 | 3004 | 344 | Not reported | 1262 (42.0) | 69 (14) | 36 (13) | 38.0 | 87.0 | 42.0* |
| Tangri | 2011 | 3449 | 386 | Not reported | 1503 (43.6) | 70 (14) | 36 (13) | 37.1 | - | 40.2* |
| Tangri 2011 validation |  | 4942 | 1177 | Not reported | 2109 (42.7) | 69 (14) | 31 (11) | 38.6 | - | 26.4* |
| Thanabalasingam | 2022 | 442 | 159 | 206 | 196 (44.3) | 73 (12) | 20 (6.2) | 56.1 | - | 54.8 |
| Thanabalasingam 4-year cohort |  | 442 | 145 | 161 |  |  |  | - | - | - |
| Thanabalasingam 2-year cohort |  | 442 | 90 | 91 |  |  |  | - | - | - |
| van den Brand | 2019 | 505 | 55 | 45 | 157 (31.1) | 58 (13) | 50 (18) | - | - | - |
| van den Brand 2019 Nephro Test cohort |  | 1385 | 72 | 94 | 443 (32.0) | 58 (15) | 51 (18) | - | - | - |
| Wang | 2019 | 17271 | 491 | 3241 | 8810 (51.0) | 75 (9) |  | 58.6 | 98.5 | 25.2 |
| Wang 2019 2 year follow up |  | 17444 | 330 | 1927 | 8773 (50.3) | 76 (9) |  | 58.9 | 98.2 | 25.3 |
| Wang | 2017 | 402 | 68 | Not reported | 169 (42.0) | 40.7 (14.1) | 82 (43.1) | - | - | - |
| Wang | 2023 | 272 | 82 | Not reported | 147 (54.0) | 60 (12.7) | 18.8 (18.0) | - | - | - |
| Wang 2023 validation of updated model |  | 117 | 33 | Not reported | 62 (53.0) | 61.2 (7.9) | 18 (17.0) | - | - | - |
| Whitlock | 2017 | 1512 | 151 | 419 | 753 (49.8) | 66.8 (13.2) | 42.3 (-) | 75.9 | - | 37.9 |
| Wu | 2022 | 90 | 26 | 8 | 49 (54.4) | 52.7 (15.9) | 29.2 (28.4) | - | - | - |
| Wu 2022 validation |  | 60 | 19 | 5 | 33 (55.0) | 53.9 (14.7) | 27.4 (27.3) | - | - | - |
| Xie | 2012 | 619 | 67 | Not reported | 305 (49.3) | 36 (12.3) | 87.9 (44.4) | - | 46.8 | - |
| Xie | 2016 | 28779 | 1730 | 7628 | 14965 (52.0) | 60 (14) | 49 (10.7) | 22.8 | 47.0 | 28.0 |
| Xu | 2021 | 1045 | 260 | Not reported | 315 (30.1) | 67.3 (13.6) | 32.95 (18.9) | 37.7 | 90.3 | 26.8 |
| Yamanouchi | 2018 | 198 | 92 | 0 | 55 (27.8) | 59 (11) | 35.2 (14.7) | 100 | - | - |
| Yamanouchi 2018 validation |  | 98 | 49 | 0 | 30 (30.6) | 61 (10) | 35.9 (15.3) | 100 | - | - |
| Yang | 2020 | 554 | 61 | 0 | 271 (48.9) | 38.1 (9.7) | 72.5 (27.5) | - | 28.5 | - |
| Yang 2020 validation |  | 392 | 37 | 0 | 193 (49.2) | 35.1 (11.5) | 81.4 (30.4) | - | 51.3 | - |
| Ye | 2022 | 1432 | 457 | 260 | 612 (42.7) | 61.6 (7.8) | 41 (13) | 100 | 93.2 | 24.3* |
| Yuan | 2020 | 1090 | 455 | Not reported | 476 (43.7) | 50.0 (11.4) | 45.6 (16.2) | 21.0 | 30.6 | 2.7 |
| Zacharias | 2022 | 4915 | 200 | Not reported | 1956 (39.8) | 60 (11.9) | 49.4 (18.2) | 26.4 | 96.1 | 22.7 |
| Zacharias 2022 CKD-REIN cohort |  | 1912 | 445 | Not reported | 637 (33.3) | 66.2 (13.0) | 34.1 (13.3) | 42.3- | 90.8 | 39.0 |
| Zacharias 2022 SKS cohort |  | 949 | 150 | Not reported | 353 (37.2) | 65.2 (14.1) | 30.8 (16.7) | 29.3 | 93.9 | 32.2 |
| Zacharias 2022 MMKD cohort |  | 202 | 75 | Not reported | 69 (34.2) | 46.3 (12.3) | 47.4 (30.0) | 0 | 89.1 | 11.9 |
| Zacharias | 2019 | 4640 | 185 | Supplementary materials but unable to access | 1814 (39.1) | 60.2 (11.9) | 49.4 (18.2) | - | - | - |
| Zhang | 2022 | 459 | 149 | 1 | 227 (49.5) |  | 32.3 (21.0) | 21.1 | 71.0 | 15.5 |
| Zhang 2022 validation |  | 326 | 25 | 0 | 140 (42.9) |  | 35.6 (20.8) | 20.6 | 72.1 | 11.0 |
| Zhang | 2020 | 1373 | 186 | Not reported | 607 (44.2) | 35.7 (13.4) | 83.3 (41.6) | - | - | - |
| Zhang | 2021 | 376 | 37 | 5 | 330 (87.8) | 31.7 (10.4) | 113.8 (30.6) | - | - | - |
| Zhang | 2021 | 135 | 62 | 5 | 38 (28.1) | 52.1 (10.4) | 48 (30.7) | 100 | 93.3 | - |
| Zhang | 2023 | 4982 | 444 | 87 | 2587 (51.9) | 37.7 (14.8) | 92.6 (28.5) | 15.3 | 41.7 | - |
| Zhu | 2020 | 992 | 449 | Not reported separately | - | - | - | - | - | - |
| Zhu | 2022 | 99 | 8 | Not reported | 47 (47.5) | 44.3 (16.3) | - | 17.2 | 61.6 | - |
| Zou | 2022 | 390 | 158 | Not reported | 117 (30.0) | 51 (9.6) | 66.6 (34.1) | 100 | 95.1 | - |

*Definitions of CVD vary across studies

**Additional file 1: Table S9. Summary of kidney failure risk prediction models and multimorbidity/frailty measures reported for renal specific disease aetiologies**

| **CKD population** | **Prediction models** | **Number of variables in the model** | **Number of external validation studies** | **Multimorbidity/frailty measure reported** |
| --- | --- | --- | --- | --- |
| IgA Nephropathy | Barbour 2019 IgA prediction tool clinical model, clinical model + MEST, full model with race | <10 | 7 |  |
|  | Berthoux ARR* |  | 4 |  |
|  | Cattran Oxford classification* |  | 2 |  |
|  | Haas classification* |  | 1 |  |
|  | Goto* |  |  |  |
|  | Keane RENAAL* |  |  |  |
|  | Kawamura Japanese histologic classification 2013 models 1-2* |  |  |  |
|  | Tanaka* |  |  |  |
|  | Barbour clinical data at biopsy model, 2-year data model, clinical data + MEST score |  | 0 |  |
|  | Chen Cox regression model |  |  |  |
|  | Pesce |  |  |  |
|  | Xie Clinical Progression Risk Score |  |  |  |
|  | Yang clinical mode, clinical model + MEST-C, clinical model + MEST-C + urinary + MMP7 |  |  |  |
|  | Barbour 2019 IgA prediction tool full model without race | 10-20 | 7 |  |
|  | Schena CDSS |  | 2 |  |
|  | Chen MLM, |  | 0 |  |
|  | Yang clinical model + MEST-C + all urinary biomarkers |  |  |  |
| AAV or Anti-GBM disease | Brix Renal risk score* | <10 | 4 |  |
|  | Wu (MPO AAV) clinical model, clinical model + MMP7, clinpath model, clinpath model + MMP7 |  | 0 |  |
| Diabetic kidney disease | Cheng clinical model, Lab model, Lab-medication model, Full model | <10 | 0 |  |
|  | Forsblom models1-2 |  |  |  |
|  | Hoshino models 1-3 |  |  |  |
|  | Jiang |  |  |  |
|  | Kwan clinical model |  |  |  |
|  | Sun clinical model, clinical-pathological model, clinical-medication model, full model |  |  |  |
|  | Yamanouchi D-score^ |  |  |  |
|  | Ye |  |  |  |
|  | Zhang DKD models 1-2 |  |  |  |
|  | Zou MLM |  |  |  |
|  | Desai TREAT ESRD model + cardiac biomarkers | 10-20 |  |  |
|  | Kwan metabolite model |  |  |  |
|  | Lin |  |  |  |
|  | Kwan clinical metabolite model | >20 |  |  |
|  | Sheer logistic regression model, LASSO model |  |  | Elixhauser comorbidity index + Physician encounters |
| Glomerular diseases | Sethi Renal Chronicity Score* | <10 |  |  |
|  | Wang models 1-2 |  |  |  |
| Lupus nephritis | Zhang LN model |  |  |  |
| FSGS | Zhu FSGS model |  |  |  |

*Original study developing model not included in the review

**Additional file 1: Table S10. Summary of PROBAST tool assessment of individual studies**

|  |  | Risk of Bias | | | | | Applicability | | | Overall | |
| --- | --- | --- | --- | --- | --- | --- | --- | --- | --- | --- | --- |
| Author | **Year** | 1. Participants | 2. Predictors | 3. Outcome | 4. Analysis | 1. Participants | | 2. Predictors | 3. Outcome | Risk of Bias | Applicability |
| Ali | 2021 | + | + | ? | ? | + | | + | + | ? | + |
| Al-Wahsh | 2021 | + | ? | - | - | + | | + | + | - | + |
| Bai | 2022 | - | + | ? | - | - | | - | + | - | - |
| Bai | 2021 | + | + | ? | - | + | | + | + | - | + |
| Barbour | 2022 | + | - | - | - | - | | + | + | - | - |
| Barbour | 2019 | - | + | - | - | - | | - | + | - | - |
| Barbour | 2016 | + | + | - | - | - | | - | + | - | - |
| Bellocchio | 2021 | - | + | ? | - | - | | + | + | - | - |
| Belur | 2020 | + | + | - | ? | - | | + | + | - | - |
| Bon | 2023 | - | ? | - | - | - | | - | + | - | - |
| Bundy | 2022 | + | + | ? | - | + | | - | + | - | - |
| Chen | 2019 | ? | ? | - | - | - | | - | + | - | - |
| Cheng | 2020 | - | ? | ? | - | - | | - | + | - | - |
| Chu | 2023 | + | ? | ? | - | + | | + | + | - | + |
| da Silva | 2023 | - | ? | ? | - | ? | | + | + | - | ? |
| da Silva | 2022 | - | ? | - | - | + | | + | + | - | + |
| Dai | 2021 | - | ? | - | - | - | | - | + | - | - |
| Desai | 2011 | + | + | ? | - | - | | + | + | - | - |
| Dimitrov | 2003 | + | + | ? | - | - | | + | + | - | - |
| Edmonston | 2019 | + | + | + | - | - | | - | + | - | - |
| Fenton | 2018 | + | + | ? | - | + | | + | + | - | + |
| Floyd | 2023 | - | ? | - | - | - | | - | + | - | - |
| Forsblom | 2014 | + | + | ? | - | - | | - | + | - | - |
| Gibertoni | 2019 | ? | + | ? | - | - | | + | + | - | - |
| Grams | 2018 | + | ? | ? | + | + | | + | + | ? | + |
| Grams | 2023 | + | ? | ? | - | + | | + | + | - | + |
| Haaskjold | 2023 | + | ? | ? | - | - | | - | + | - | - |
| Hallan | 2019 | + | + | ? | - | - | | + | + | - | - |
| Hasengawa | 2019 | - | + | ? | ? | - | | - | + | ? | - |
| Hoshino | 2015 | - | ? | ? | - | - | | - | + | - | - |
| Hsu | 2017 | + | + | - | - | + | | - | + | - | - |
| Hundemer | 2020 | - | ? | ? | - | + | | + | + | - | + |
| Hwang | 2021 | + | ? | - | - | - | | - | + | - | - |
| Ingwiller | 2022 | - | - | ? | - | + | | + | + | - | + |
| Irish | 2023 | + | ? | ? | - | + | | + | + | - | + |
| Jahan | 2023 | + | ? | ? | - | + | | + | + | - | + |
| Jiang | 2019 | - | + | ? | - | - | | - | + | - | - |
| Johnson | 2008 | + | ? | ? | - | + | | + | + | - | + |
| Johnson | 2007 | + | ? | ? | - | + | | + | + | - | + |
| Kang | 2020 | - | ? | ? | - | + | | + | + | - | + |
| Knoop | 2015 | ? | ? | ? | - | - | | - | - | - | - |
| Kong | 2023 | - | ? | ? | - | - | | - | + | - | - |
| Kwan | 2020 | + | + | ? | - | - | | + | + | - | - |
| Kwek | 2022 | - | ? | ? | - | + | | + | + | - | + |
| Landray | 2010 | + | + | ? | - | + | | + | + | - | + |
| Lee | 2018 | - | - | ? | - | + | | + | + | - | + |
| Lennartz | 2016 | - | + | ? | - | ? | | + | + | - | - |
| Lim | 2019 | - | + | + | - | + | | + | + | - | + |
| Lin | 2023 | ? | + | - | - | - | | + | + | - | - |
| Maher | 2023 | + | ? | ? | + | + | | + | + | ? | + |
| Major | 2019 | - | ? | ? | - | + | | + | + | - | + |
| Massy | 2023 | + | + | + | - | + | | - | + | - | - |
| Maziarz | 2015 | ? | ? | - | - | + | | + | + | - | + |
| Maziarz | 2014 | ? | ? | + | - | + | | + | + | - | + |
| Naranjo | 2021 | ? | + | - | - | + | | ? | + | - | + |
| Orlandi | 2018 | + | + | - | - | + | | + | + | - | + |
| Ouyang | 2021 | - | ? | - | - | - | | - | + | - | - |
| Park | 2014 | - | ? | - | - | - | | - | - | - | - |
| Peeters | 2013 | - | + | ? | - | + | | + | + | - | + |
| Pesce | 2016 | - | ? | ? | - | - | | - | + | - | - |
| Prouvot | 2021 | + | + | ? | - | + | | + | + | - | + |
| Ramspek | 2021 | + | - | ? | ? | + | | + | + | - | + |
| Sato | 2015 | - | ? | - | - | - | | - | - | - | - |
| Schena | 2021 | - | ? | - | - | - | | - | + | - | - |
| Schroeder | 2017 | + | + | + | - | + | | + | + | - | + |
| Sheer | 2022 | - | + | - | - | - | | + | + | - | - |
| Smith | 2013 | ? | + | ? | - | + | | - | - | - | - |
| Stefan | 2020 | - | + | ? | - | - | | - | + | - | - |
| Sud | 2014 | ? | - | ? | - | + | | - | + | - | - |
| Sun | 2020 | + | + | ? | - | - | | - | + | - | - |
| Tangri | 2016 | + | ? | ? | - | + | | + | + | - | + |
| Tangri | 2017 | + | ? | ? | - | + | | + | + | - | + |
| Tangri | 2011 | + | ? | ? | - | + | | + | + | - | + |
| Thanabalasingam | 2022 | + | ? | ? | - | + | | + | + | - | + |
| van den Brand | 2019 | - | + | - | - | + | | + | + | - | + |
| Wang | 2019 | - | + | ? | - | - | | - | + | - | - |
| Wang | 2017 | + | ? | - | - | + | | - | + | - | - |
| Wang | 2023 | + | ? | - | - | + | | + | + | - | + |
| Whitlock | 2017 | + | ? | ? | - | + | | + | + | - | + |
| Wu | 2022 | ? | + | + | - | - | | - | + | - | - |
| Xie | 2012 | - | ? | ? | - | - | | + | + | - | - |
| Xie | 2016 | + | ? | ? | - | + | | + | + | - | + |
| Xu | 2021 | ? | ? | - | - | ? | | + | + | - | ? |
| Yamanouchi | 2018 | + | ? | ? | - | - | | - | + | - | - |
| Yang | 2020 | + | + | + | - | - | | - | + | - | - |
| Ye | 2022 | ? | + | + | + | - | | + | + | ? | - |
| Yuan | 2020 | - | ? | ? | - | + | | + | + | - | + |
| Zacharias | 2022 | + | + | + | - | + | | - | + | - | - |
| Zacharias | 2019 | + | + | + | + | + | | - | + | + | - |
| Zhang | 2022 | + | + | - | - | - | | - | + | - | - |
| Zhang | 2020 | + | + | - | - | - | | - | + | - | - |
| Zhang | 2021 | + | + | - | - | - | | - | + | - | - |
| Zhang | 2021 | + | ? | ? | - | + | | + | + | - | + |
| Zhang | 2023 | - | + | + | - | - | | - | + | - | - |
| Zhu | 2020 | - | + | + | - | - | | + | + | - | - |
| Zhu | 2022 | - | ? | + | - | - | | - | + | - | - |
| Zou | 2022 | + | ? | - | - | - | | - | + | - | - |

**Additional file 1: Table S11. Competing risk of death consideration and performance measures of included studies**

| Author | Year | Discrimination measure | Calibration measure | Competing risk of death considered |
| --- | --- | --- | --- | --- |
| Ali | 2021 | AUC | Calibration plot | Yes |
| Al-Wahsh | 2021 | c-index | Calibration plot, Brier score | Yes |
| Bai | 2022 | AUC | - | No |
| Bai | 2021 | AUC | - | Yes |
| Barbour | 2022 | c-index | Calibration curves/slopes | No |
| Barbour | 2019 | c-index | Calibration plots | No |
| Barbour | 2016 | c-index | Calibration plots | No |
| Bellocchio | 2021 | AUC | Calibration plots | No |
| Belur | 2020 | AUC | Calibration plots | No |
| Bon | 2023 | AUC | - | No |
| Bundy | 2022 | AUC | Calibration plots | No |
| Chen | 2019 | c-index | O:E by deciles, Hosmer-Lemeshow test | No |
| Cheng | 2020 | c-index | Calibration curves, Hosmer-Lemeshow test | No |
| Chu | 2023 | c-index | - | No |
| da Silva | 2023 | AUC | Hosmer-Lemeshow test | No |
| da Silva | 2022 | AUC | Hosmer-Lemeshow test | No |
| Dai | 2021 | AUC | O:E by deciles | No |
| Desai | 2011 | c-index | - | Yes |
| Dimitrov | 2003 | AUC | - | No |
| Edmonston | 2019 | AUC | - | No |
| Fenton | 2018 | c-index | Hosmer-Lemeshow test | No |
| Floyd | 2023 | c-index | - | No |
| Forsblom | 2014 | AUC, c-index | R2 | Yes |
| Gibertoni | 2019 | Visual inspection of K-M curves | Calibration plots | Yes |
| Grams | 2018 | c-index | R2 | Yes |
| Grams | 2023 | c-index | Calibration slope | Yes |
| Haaskjold | 2023 | AUC | Calibration plots | No |
| Hallan | 2019 | AUC | Calibration curves, Hosmer-Lemeshow test | Compared to risk of death using MREK |
| Hasengawa | 2019 | Time varying AUC | - | Yes |
| Hoshino | 2015 | c-index | - | No |
| Hsu | 2017 | c-index | - | No |
| Hundemer | 2020 | AUC | Calibration curves, Hosmer-Lemeshow test, Brier score | Yes |
| Hwang | 2021 | AUC | Calibration plots | No |
| Ingwiller | 2022 | AUC | Hosmer-Lemeshow test | No |
| Irish | 2023 | c-index | O:E, Brier score | No |
| Jahan | 2023 | AUC | O:E | Yes |
| Jiang | 2019 | c-index | Calibration plot | No |
| Johnson | 2008 | c-index | calibration quintiles/curves, Hosmer-Lemeshow test | No |
| Johnson | 2007 | c-index |  | No |
| Kang | 2020 | Time dependent AUC | calibration plot, Brier score | No |
| Knoop | 2015 | AUC | - | No |
| Kong | 2023 | Time dependent AUC | Calibration plot | No |
| Kwan | 2020 | AUC | - | Yes |
| Kwek | 2022 | c-index | Calibration curves | No |
| Landray | 2010 | AUC | O:E | No |
| Lee | 2018 | c-index | Brier score | No |
| Lennartz | 2016 | c-index | Calibration plots | No |
| Lim | 2019 | c-index | Nam-D'Agostino X2 statistic, Brier score | No |
| Lin | 2023 | c-index | Brier score | No |
| Maher | 2023 | c-index | O:E, calibration plots, intercept/slope | Yes |
| Major | 2019 | c-index, Somer’s D | Calibration plots | No |
| Massy | 2023 | c-index | O:E absolute risk | Yes |
| Maziarz | 2015 | AUC | Prediction error | No |
| Maziarz | 2014 | AUC | Prediction error | No |
| Naranjo | 2021 | c-index | Calibration plots, Greenwood-Nam-D'Agostino statistic | No |
| Orlandi | 2018 | c-index | Calibration plot | No |
| Ouyang | 2021 | c-index | Calibration curves, Hosmer-Lemeshow test | No |
| Park | 2014 | c-index | - | No |
| Peeters | 2013 | AUC | Calibration plots, Hosmer-Lemeshow test | No |
| Pesce | 2016 | AUC | - | No |
| Prouvot | 2021 | AUC | Calibration plot, Hosmer-Lemeshow test | No |
| Ramspek | 2021 | c-index | Calibration plots | Yes |
| Sato | 2015 | - | Hosmer-Lemeshow test | N/A (no recorded deaths) |
| Schena | 2021 | c-index | May-Hosmer test | No |
| Schroeder | 2017 | c-index | Calibration curves | No |
| Sheer | 2022 | c-index | - | No |
| Smith | 2013 | c-index | R2 | No |
| Stefan | 2020 | AUC | Hosmer-Lemeshow test | No |
| Sud | 2014 | c-index | X2 statistic Nam and D’Agostino | Yes |
| Sun | 2020 | c-index | Hosmer-Lemeshow test | No |
| Tangri | 2016 | c-index | Calibration plots (quintiles), Brier score | No |
| Tangri | 2017 | c-index | Calibration curves/deciles, X2 statistic (Nam and D’Agostino) | No |
| Tangri | 2011 | c-index | Calibration plots, X2 statistic (Nam and D’Agostino) | Yes |
| Thanabalasingam | 2022 | AUC, c-index | Calibration plots | Yes for Grams |
| van den Brand | 2019 | AUC | Calibration curves | No |
| Wang | 2019 | AUC | Brier | Yes |
| Wang | 2017 | AUC | - | No |
| Wang | 2023 | c-index | O:E by quintiles, Hosmer- Lemeshow test | No |
| Whitlock | 2017 | c-index | - | No |
| Wu | 2022 | c-index | R2 | No |
| Xie | 2012 | AUC | R2 | No |
| Xie | 2016 | AUC | Prediction error | No |
| Xu | 2021 | AUC | Calibration curve | No |
| Yamanouchi | 2018 | AUC | - | N/A (no recorded deaths) |
| Yang | 2020 | c-index | - | N/A (no recorded deaths) |
| Ye | 2022 | c-index | Calibration plot | Yes |
| Yuan | 2020 | AUC | - | No |
| Zacharias | 2022 | c-index | Calibration curves | Yes |
| Zacharias | 2019 | c-index | - | Yes |
| Zhang | 2022 | c-index | Calibration curve | N/A composite outcome of death |
| Zhang | 2020 | c-index | Calibration slope | No |
| Zhang | 2021 | AUC | - | N/A composite outcome of death |
| Zhang | 2021 | c-index | Calibration plot | N/A composite outcome of death |
| Zhang | 2023 | AUC, c-index | Calibration plots | No |
| Zhu | 2020 | AUC | Brier | N/A composite outcome of death |
| Zhu | 2022 | c-index | Calibration plots | No |
| Zou | 2022 | AUC | - | No |

AUC – area under the receiver operating characteristic curve

O:E – observed versus predicted
