# Supplementary material for: Representation of multimorbidity and frailty in the development and validation of kidney failure prognostic prediction models: a systematic review
Source: BMC Med. 2024 Oct 11;22:452. doi: 10.1186/s12916-024-03649-9 (PMC11470573; doi:10.1186/s12916-024-03649-9)
Supplement: Supplementary file 2 — Additional file 2: Figure S1. Baseline co-morbidities recorded and included/added to externally validated kidney failure prediction models for renal specific disease aetiologies. [file 12916_2024_3649_MOESM2_ESM.docx]

**Additional file 2**

**Additional file 2: Figure S1. Baseline co-morbidities recorded and included/added to externally validated kidney failure prediction models for renal specific disease aetiologies**

|  | | **Comorbidities recorded at baseline or included/added to model** | | | |
| --- | --- | --- | --- | --- | --- |
| **Model** | **Study** | **Hypertension** | **Diabetes** | **CV disease** | **Other** |
| Oxford Classification | Park 2014 | 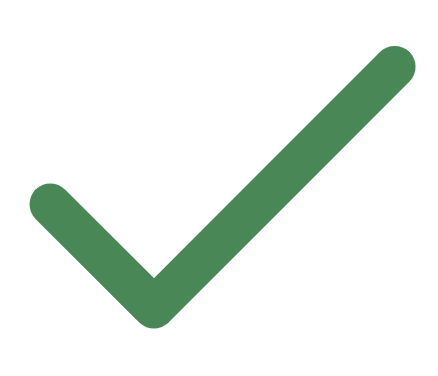 |  |  |  |
|  | Hwang 2021 | 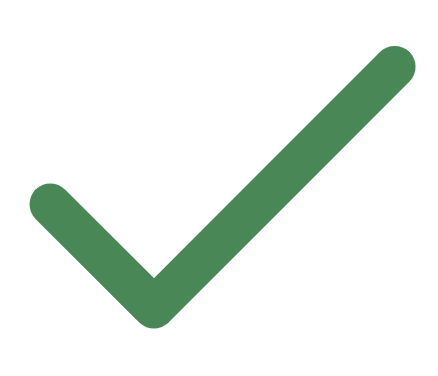 | 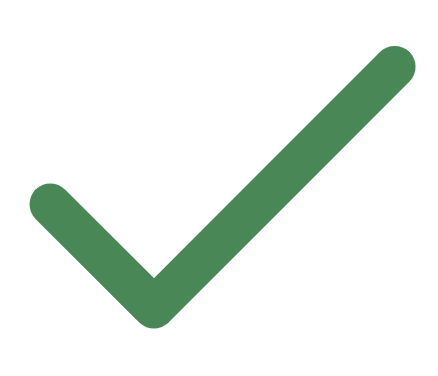 |  |  |
| Barbour IgA prediction tool |  | 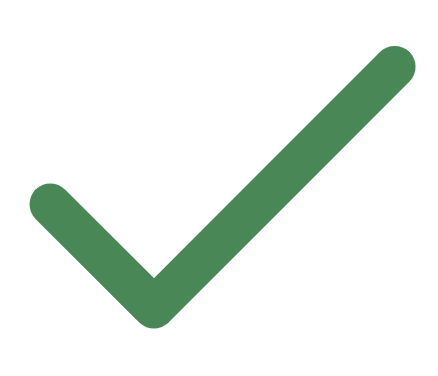 | 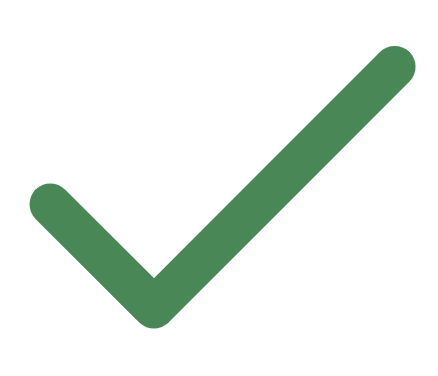 |  |  |
|  | Schena 2021 | 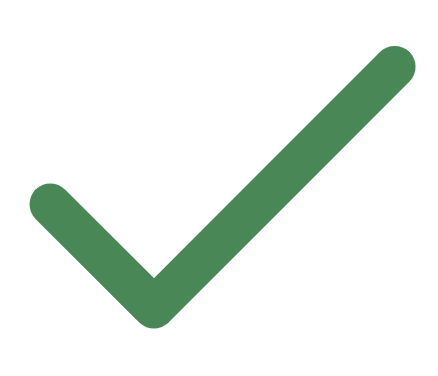 |  |  |  |
| Berthoux ARR | Knoop 2015 | 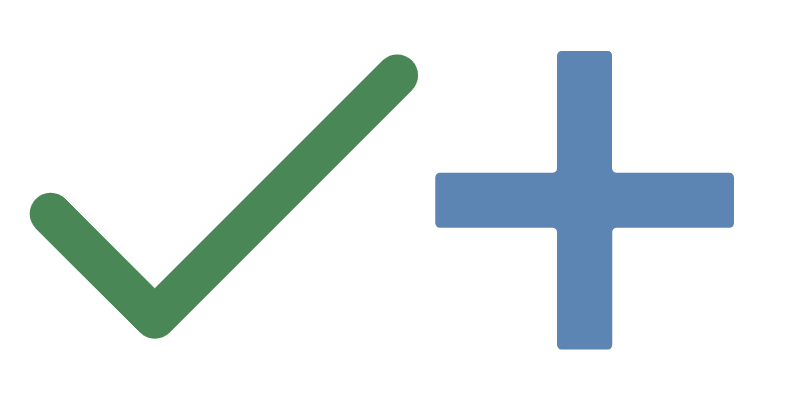 |  |  |  |
|  | Pesce 2016 | 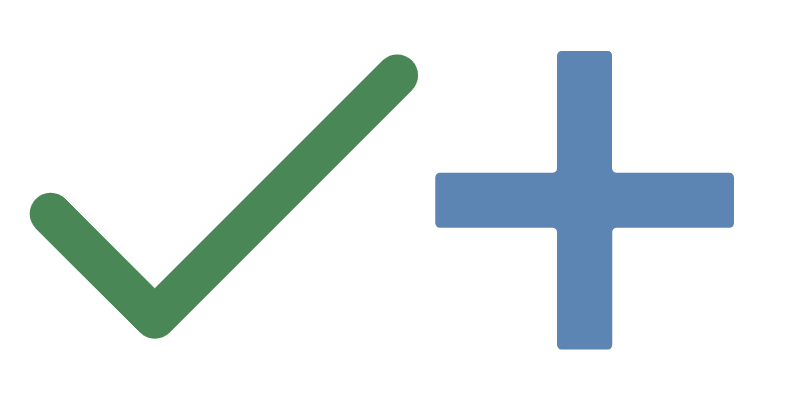 |  |  |  |
|  | Schena 2021 | 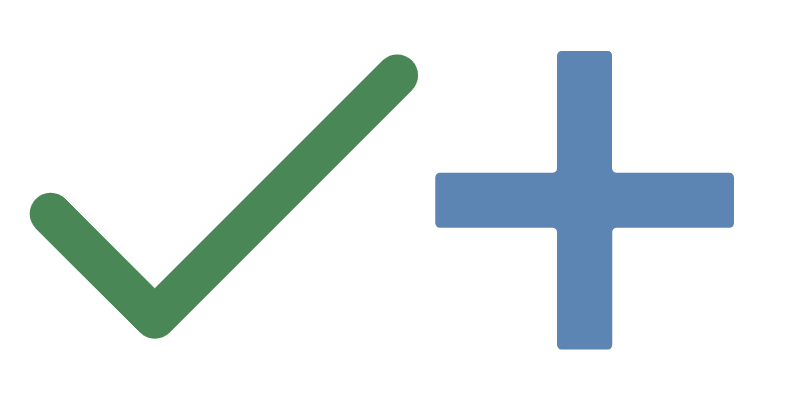 |  |  |  |
|  | Xie 2012 | 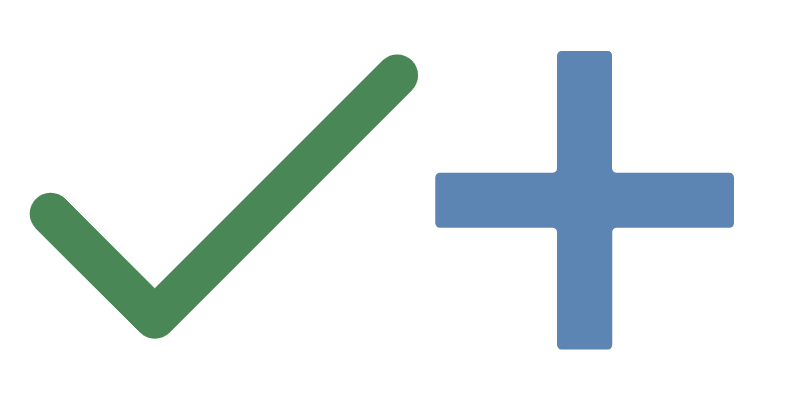 |  |  |  |
| Brix RRS | Kong 2023 | 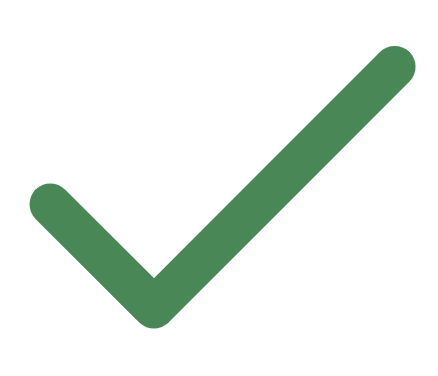 | 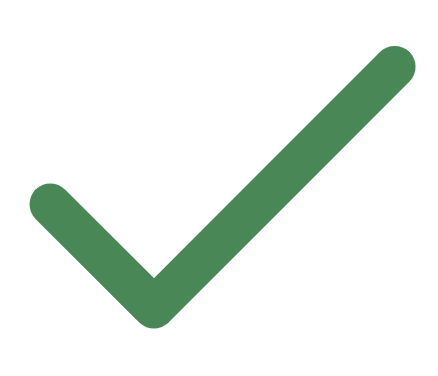 |  |  |
| Haas classification | Park 2014 | 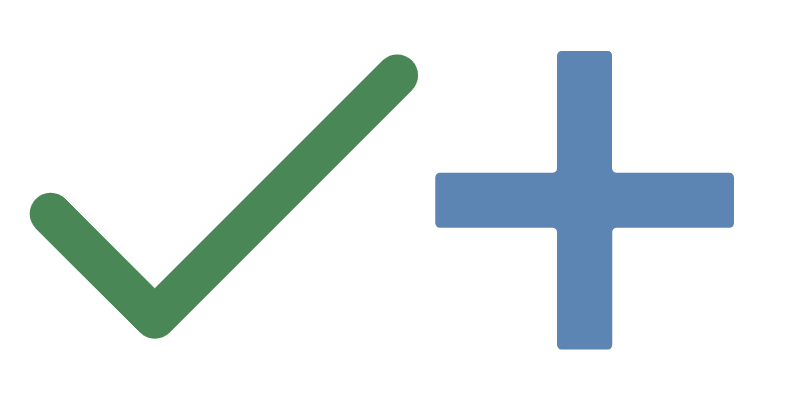 |  |  |  |
| Goto | Xie 2012 | 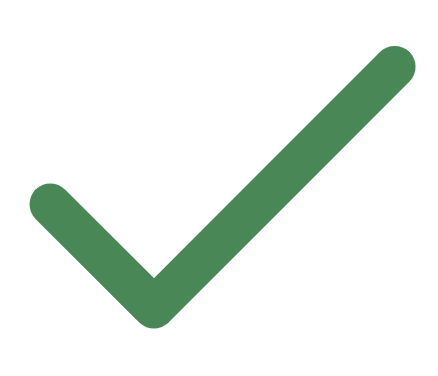 |  |  |  |
| Keane RENAAL |  | 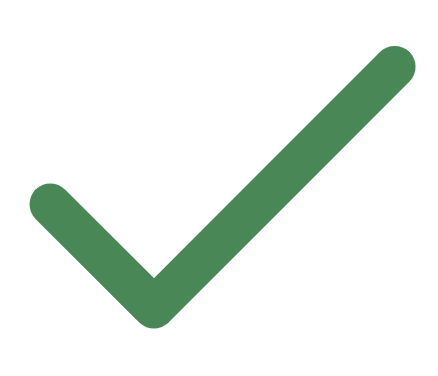 |  |  |  |

| 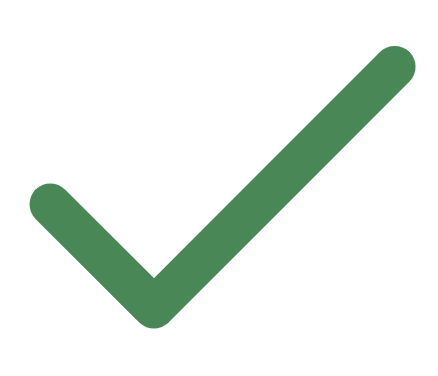 | Recorded at baseline |
| --- | --- |
| 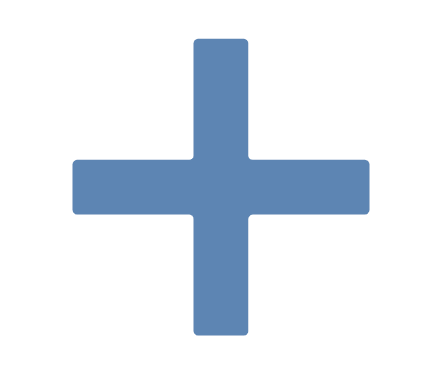 | Included in model |
